# Machine Learning for Localizing Epileptogenic-Zone in the Temporal Lobe: Quantifying the Value of Multimodal Clinical-Semiology and Imaging Concordance

**DOI:** 10.3389/fdgth.2021.559103

**Published:** 2021-02-10

**Authors:** Ali Alim-Marvasti, Fernando Pérez-García, Karan Dahele, Gloria Romagnoli, Beate Diehl, Rachel Sparks, Sebastien Ourselin, Matthew J. Clarkson, John S. Duncan

**Affiliations:** ^1^Department of Clinical and Experimental Epilepsy, UCL Queen Square Institute of Neurology, University College London, London, United Kingdom; ^2^Department of Medical Physics and Biomedical Engineering, University College London, London, United Kingdom; ^3^Wellcome/EPSRC Centre for Interventional and Surgical Sciences (WEISS), London, United Kingdom; ^4^National Hospital for Neurology and Neurosurgery, London, United Kingdom; ^5^School of Biomedical Engineering & Imaging Sciences (BMEIS), King's College London, London, United Kingdom; ^6^University College London Medical School, London, United Kingdom

**Keywords:** epilepsy surgery, machine learning, semiology, hippocampal sclerosis, epileptogenic zone, temporal lobe epilepsy, gradient boost classifier, linear support vector classifier

## Abstract

**Background:** Epilepsy affects 50 million people worldwide and a third are refractory to medication. If a discrete cerebral focus or network can be identified, neurosurgical resection can be curative. Most excisions are in the temporal-lobe, and are more likely to result in seizure-freedom than extra-temporal resections. However, less than half of patients undergoing surgery become entirely seizure-free. Localizing the epileptogenic-zone and individualized outcome predictions are difficult, requiring detailed evaluations at specialist centers.

**Methods:** We used bespoke natural language processing to text-mine 3,800 electronic health records, from 309 epilepsy surgery patients, evaluated over a decade, of whom 126 remained entirely seizure-free. We investigated the diagnostic performances of machine learning models using set-of-semiology (SoS) with and without hippocampal sclerosis (HS) on MRI as features, using STARD criteria.

**Findings:** Support Vector Classifiers (SVC) and Gradient Boosted (GB) decision trees were the best performing algorithms for temporal-lobe epileptogenic zone localization (cross-validated Matthews correlation coefficient (MCC) SVC 0.73 ± 0.25, balanced accuracy 0.81 ± 0.14, AUC 0.95 ± 0.05). Models that only used seizure semiology were not always better than internal benchmarks. The combination of multimodal features, however, enhanced performance metrics including MCC and normalized mutual information (NMI) compared to either alone (*p* < 0.0001). This combination of semiology and HS on MRI increased both cross-validated MCC and NMI by over 25% (NMI, SVC SoS: 0.35 ± 0.28 vs. SVC SoS+HS: 0.61 ± 0.27).

**Interpretation:** Machine learning models using only the set of seizure semiology (SoS) cannot unequivocally perform better than benchmarks in temporal epileptogenic-zone localization. However, the combination of SoS with an imaging feature (HS) enhance epileptogenic lobe localization. We quantified this added NMI value to be 25% in absolute terms. Despite good performance in localization, no model was able to predict seizure-freedom better than benchmarks. The methods used are widely applicable, and the performance enhancements by combining other clinical, imaging and neurophysiological features could be similarly quantified. Multicenter studies are required to confirm generalizability.

**Funding:** Wellcome/EPSRC Center for Interventional and Surgical Sciences (WEISS) (203145Z/16/Z).

## Introduction

Fifty million people have epilepsy world-wide, and one third are refractory to two or more appropriate antiepileptic drugs, with recurrent seizures and impairment of quality of life. Neurosurgical resections in focal epilepsy may be curative and have been shown to improve health status (1–3). The Epileptogenic Zone (EZ) is defined as the region that when resected, renders the patient seizure-free. Understanding the symptoms, signs and semiology (chronological clinical seizure manifestations) at the onset of seizures is key to determining the site of seizure onset in the brain; but this may be imprecise (4). Despite an extensive literature on semiology, imaging and electroencephalographic (EEG) features for EZ-localization, no definitive method exists to determine the EZ (5). Concordance is sought with brain imaging: MRI, functional imaging (SPECT, FDG-PET); scalp EEG video-telemetry and neuropsychology. The results are discussed in a multidisciplinary team (MDT) conference, to localize the EZ and minimize risks, prior to consideration of resection. Despite this, many patients do not become seizure-free after surgery (6).

The value of any particular clinical feature or investigation result in contributing to a patient's differential diagnosis depends on its overall univariate association with the EZ (prior) and any other factors which may interact with it. Clinical judgement and acumen arise through experience, when there may not be objective data. Although one can assess the value of clinical features through Bayesian-belief elicitation, in the absence of grounded-objectives, responses would be capturing subjective clinical values (7). Well-designed machine learning methods using ground-truth target labels and all relevant features perform well in capturing data patterns to predict targets, akin to clinical intuition. The so-called “AI chasm” notes that algorithms are only clinically useful if they improve clinical outcomes, not just diagnostic accuracy (8).

A study in 2015 evaluated 830 patients and the value of semiology in predicting the EZ (9). Conditional inference trees' localization accuracy among five ictal onset areas was 56.1%. Accuracy for binary mesial temporal lobe epilepsy (mTLE) or lateral temporal-EZ was 71% (unquoted naïve accuracy of 63%) (9). Despite the large numbers, the supervised learning method suffered from inadequate ground-truth labels: the EZ was often labeled by clinicians on the presence or absence of a particular semiology, making the evaluation logic circular and results were reported without cross-validation or test sets, compromising generalizability. A review in 2017 showed algorithmic identification of EZ brain networks and the propagation of seizures remains an open issue. Combinations of multimodal features have not been used on large-scale high-quality patient data (10). Currently there are no clinically utilized algorithms to augment EZ-localization or quantify the value of multimodal features presented in MDTs.

In this study, we set out to objectively assess the value of combining clinical features for temporal-lobe (TL) epileptogenic zone localization – the most common form of drug refractory epilepsy with the best surgical outcomes. We investigated set of seizure semiology (SoS, devoid of sequence information) and hippocampal sclerosis (HS), as this imaging finding is specific to the TL, is the most frequent imaging finding, and provides a good univariate benchmark. HS is a scar in the medial temporal lobe and the most common pathology underlying drug-resistant TL epilepsy. These features are important in clinical evaluations and can be extracted from electronic health record texts. We used machine learning models with strong ground-truths and also assessed values in predicting surgical outcomes.

## Methods

### Study Design and Participants

Our objective was to determine the value of clinical-semiology, hippocampal sclerosis and their combination for the binary localization of the EZ to the temporal or extratemporal brain. The value of combining these features was quantified for both relative diagnostic performance (Step 1) and subsequently using the model from Step 1 for post-surgical prognosis (Step 2) as well as training independent models for the direct prediction of surgical outcomes (Step 3).

Retrospective text analysis of 3,800 mixed data-type electronic health records (EHRs) pertaining to adults with refractory focal epilepsy admitted for presurgical assessment for epilepsy surgery from 2001 to 2011 was undertaken at the National Hospital for Neurology and Neurosurgery, London. SoS, HS, and temporal-EZ features were extracted (Table 1). Univariate statistics were computed and machine learning models were trained to predict temporal-EZ and subsequently prognosis.

**Table 1:** Frequency of Features and Targets.

**Variable**	**Frequency in seizure-free patients (*n* = 126) (%)**	**Frequency in all operated patients (*n* = 309) (%)**
Temporal-EZ (target)	112 (89%)	256 (mix of seizure-free and not seizure-free) (83%)
Dialeptic/loss of awareness (LOA)	92 (73%)	223 (72%)
Tonic-clonic	84 (67%)	224 (72%)
Hippocampal sclerosis (imaging feature)	70 (56%)	147 (48%)
Oral automatisms	58 (46%)	140 (45%)
Other automatism (unspecified)	57 (45%)	138 (45%)
Olfactory-gustatory	56 (44%)	141 (46%)
Upper limb automatism	49 (39%)	108 (35%)
Tonic	47 (37%)	126 (41%)
Aphasia	46 (37%)	100 (32%)
Fear-Anxiety	37 (29%)	91 (29%)
Head Turn	30 (24%)	73 (24%)
Clonic	30 (24%)	77 (25%)
Epigastric	28 (22%)	61 (20%)
Autonomous-vegetative	26 (21%)	66 (21%)
Psychic	23 (18%)	57 (18%)
Non-specific aura	22 (17%)	52 (17%)
Dysphasia	21 (17%)	71 (23%)
LOC	17 (13%)	46 (15%)
Astatic	15 (12%)	38 (12%)
Other simple motor	14 (11%)	32 (10%)
Vocalization	13 (10%)	33 (11%)
Somatosensory	12 (10%)	39 (13%)
Nose-wiping	10 (8%)	18 (6%)
Dystonic	10 (8%)	26 (8%)
Head version	10 (8%)	27 (9%)
Grimace	10 (8%)	19 (6%)
Blink	9 (7%)	27 (9%)
Hypermotor	8 (6%)	19 (6%)
Dacrystic	8 (6%)	14 (5%)
Vestibular	7 (6%)	26 (8%)
Other complex motor	6 (5%)	13 (4%)
Auditory	4 (3%)	10 (3%)
Gelastic	4 (3%)	7 (2%)
Eye Version	3 (2%)	8 (3%)
Hypomotor (behavioral arrest)	3 (2%)	11 (4%)
Visual	3 (2%)	12 (4%)
Coprolalia	3 (2%)	3 (1%)
Figure of 4	2 (2%)	5 (2%)
Atonic	2 (2%)	6 (2%)
Ictal pout	1 (1%)	1 (0.3%)
Myoclonic	1 (1%)	2 (1%)
Spitting	1 (1%)	7 (2%)
Asymmetric tonic	1 (1%)	4 (1%)
Fencing	0	1 (0.3%)
Lower limb automatism	0	1 (0.3%)
Palilalia	0	0
Aphemia	0	0
Drinking	0	0
Cough	0	0
Whistling	0	0

*Frequency of patients with Semiology, imaging feature and temporal resections. By “hypomotor” we mean behavioral arrest during a seizure and not the semiology specific to the pediatric population*.

We used set-of-semiology (SoS), because these are more readily available from a clinical history than precise symptom chronology. We restricted MRI-identifiable TL pathology to HS as this represented 92% of temporal lesions (*n* = 70).

### Procedures

EHRs were pseudo-anonymised, pre-processed and text-mined for the presence of 49 semiology features and a single imaging feature (HS) using regular expressions as a taxonomy replacement. This taxonomy replacement was a bespoke expansion of major semiological categories presented elsewhere (4). The anonymised keys and identifiers were stored in secure NHS systems and checks for data-mining integrity on a subsample showed <5% binary-feature error compared to manual feature-extraction by a consultant neurologist. The Pandas DataFrame was sparse and multi-one-hot encoded. EHRs were cross-referenced to a database containing EZ-localization labels (resected lobes) alongside their post-operative year-by-year ordinal score on the ILAE epilepsy surgery outcome scale, and whether they had intracranial electrode recordings, curated since 1990, as previously reported (6). Intracranial electrodes were collected only as a univariate benchmark for negative prognostic value in epilepsy surgery, as their presence is a clinical indicator of uncertain EZ.

EHRs from 870 cases were available, 335 of which underwent epilepsy-surgery after assessment. 324 cases were from unique patients, of which 309 had one resection only, excluding hemispherectomies and corpus callosotomies, consistent with previous methodology (11).

### Statistical Analysis

Fisher's exact and Mann-Whitney *U*-tests were performed at three levels of uncorrected type I error (alpha = 0.05, 0.005, and 0.0005) with Bonferroni corrections for multiple comparisons for 181 tests (Fisher's: 51 for Step 1, 53 × 2 for Step 3; MWU: 24 tests) (*p* < 2.76 × 10^−4^ = ^*^, *p* < 2.76 × 10^−5^ = ^**^, *p* < 2.76 × 10^−6^ = ^***^, respectively). Theil's U (asymmetric normalized mutual information, NMI) was used to check for categorical correlations and model performance.

### Machine Learning

We used multivariate binary Logistic Regression (LR), Gradient Boosted Trees (GB), and Linear Support Vector Classifiers (SVC) (implemented in Scikit-learn v 0.19.2) (12) as suggested by previous studies (9, 13). We chose these specific algorithms as LR is widely used in predictive models, SVC performs well if the target can be linearly separated by a high-dimensional hyperplane in feature space, and GB ensemble models leverage multiple weak classifiers into a strong classifier with each individual component utilizing a different feature subset, akin to clinical MDTs. GB are more likely to succeed with more data and complexity, but are less interpretable than SVC or LR. For binary features and binary outcomes as in our study, LR without regularization can have a decision boundary that asymptotically approaches that of SVC (14), which can further help assess if the targets are linearly separable. Feature selection was performed using both univariate and recursive feature elimination with 5-fold cross-validation (RFECV) methods (15). No other hyperparameter tuning was performed.

The models were compared to benchmarks in localizing temporal-EZ (Step 1). We also made indirect assessments if improved diagnostic accuracy translated to enhanced outcome predictions (Step 2), and separately trained models to directly predict outcomes (Step 3). For Step 1, we chose a binary localization target containing the most common focal epilepsy, temporal-lobe vs. extra-temporal (ET) EZ, and models were trained on patients who were entirely seizure-free at all follow-up years (ESF). For Steps 2 and 3, outcome was assessed at two binary levels: seizure-freedom at 1-year (ILAE1), and ESF. In Step 2, the Step 1 model was used to predict outcomes on all data. In Step 3, new models were trained to predict outcomes. ILAE 2 and above were considered not seizure-free (NSF) due to residual epileptogenic tissue resulting in auras or seizures with impaired awareness.

Although we report many metrics (using 1,000 × 5 repeated stratified CV with means and standard deviations in **Table 3**, or medians and IQR), due to an unbalanced dataset, we focus on Matthews-correlation-coefficient (MCC) as one of the most suitable metrics for binary classification evaluations which can be interpreted as a discretization of Pearson's-correlation-coefficient (16, 17). NMI was used to quantify information gains between features, models, and the ground truth EZ.

### Role of the Funding Source

The Wellcome/EPSRC Center for Interventional and Surgical Sciences had no role in the study design; collection, analysis or interpretation of data; writing of report; nor in the decision to submit for publication.

This study was approved by the Research Ethics Committee for UCL and UCLH (20/LO/0149).

## Results

### Patients and Outcomes

Of the 309 patients, 126 (41%) were ESF at all follow-up years (median follow-up 7 years, IQR = 5–10, Supplementary Figure 9), indicating correct EZ-resections. Labels were unbalanced; 112/126 (88.9%) were temporal-EZ, and 14 extratemporal.

### Features

Forty-two semiology features were present in the ESF-set. Automatisms (oral, manual and other) were merged to a single category, leaving 40 SoS features. There were 76 temporal-lobe lesions in the ESF group and HS as the single imaging feature constituted 92% (70/76) of these. In addition, there were three cavernomas, one dysembryoplastic neuroepithelial tumor, one cyst and one focal cortical dysplasia in the temporal lobes.

Table 1 shows frequency of occurrences in the 126-ESF-set and all 309 operated patients.

Table 2 shows univariate benchmarks for features associated with temporal-EZ. The statistically significant features after multiple-comparisons correction on two-by-two Fisher's exact tests were seizures with automatisms and HS. The highest odds-ratios were for presence of HS, automatisms, and fear-anxiety. The performance metrics of the best univariate features, as benchmarks, are summarized in Table 3.

**Table 2:** Benchmarks for Step 1 Temporal-EZ Localization.

**Feature**	**Number with TL-EZ/number with feature (*n* = 126)**	**Number with TL-EZ/number with feature (*n* = 309)**	**Odds ratios (*n* = 126, *n* = 309)**	***p*-values (*n* = 126, *n* = 309)**
**Temporal-EZ features**				
Hippocampal sclerosis	70/70	144/147	DBZ^**^, 21^***^	4.2 × 10^−6^^**^, 6.3 × 10^−13^^***^
All Automatisms (combined)	82/84	186/206	16.4^*^, 4.4^***^	3.0 × 10^−5^^*^, 2.2 × 10^−6^^***^
Oral automatisms	58/58	131/140	DBZ^*^, 5.1^**^	9.7 × 10^−5^^*^, 3.5 × 10^−6^^**^
Other automatism (unspecified)	55/57	127/138	5.8, 3.8^*^	0.020, 0.00012^*^
Upper limb automatism	49/49	100/108	DBZ, 3.6	0.00082, 0.00077
Fear-anxiety	37/37	84/91	DBZ, 3.2	0.010, 0.0045
Dialeptic/LOA	85/92	195/223	3.1, 2.9	0.054, 0.0012
Epigastric	NS	58/61	NS, 4.9	NS, 0.0039
Aphasia	NS	90/100	NS, 2.3	NS, 0.024
**Extratemporal-EZ features**				
Intracranial electrodes	NS	50/89	NS, 0.09	NS, 7.1 × 10^−4^
Hypomotor (behavioral arrest)	0/3	6/11	0, 0.16	0.0011, 0.0045
Somatosensory	8/12	25/39	0.19, 0.30	0.029, 0.0024
Clonic	23/30	57/77	0.26, 0.47	0.040, 0.023
Head version	NS	16/27	NS, 0.25	NS, 0.0021
Eye version	NS	3/8	NS, 0.11	NS, 0.0046
Asymmetric tonic	NS	1/4	NS, 0.07	NS, 0.017

*Fisher's exact test for Step 1 Temporal-EZ localization in postoperative seizure-free patients (n = 126, strong ground truths) and all operated patients (n = 309, 256 weakly labeled as temporal, 53 as extratemporal). All features with p < 0.05 are shown;*

* *Represents significance at alpha 5% after Bonferroni correction*.

** *at 0.5% after Bonferroni correction*.

*** *at 0.05% after Bonferroni correction. DBZ, Division By Zero. NS: p > 0.05*.

**Table 3:**
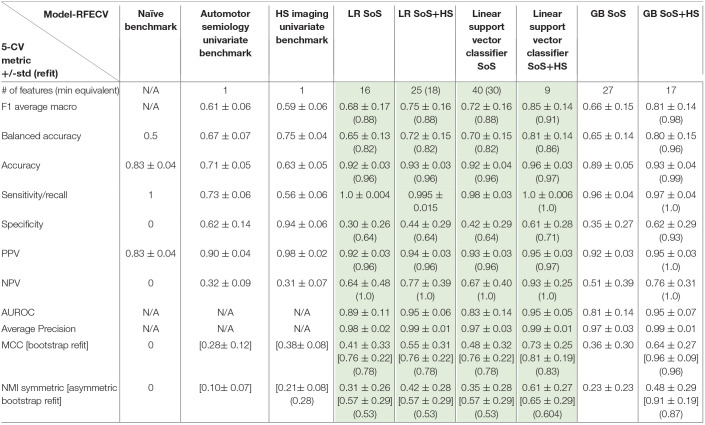
Machine Learning Models for Temporal EZ-Localization (Step 1).

*Step 1 CV performance metrics. Mean and standard deviation of 1,000 × 5 CV scores. Benchmark std given by bootstrapping 2,000 × 5 CV. Brackets represent model-refit (training) scores. Square brackets show bootstrapped refit results. CV, cross-validation; RFECV, Recursive Feature Elimination with CV; std, standard deviation; PPV/NPV, Positive/Negative Predictive Value; AUROC, Area under receiver operating curve; MCC, Matthews Correlation Coefficient; NMI, Normalized Mutual Information. See Supplementary Materials for expanded table and distribution of MCC and NMI scores*.

### Step 1: EZ Cross-Validated Results

**Figure 1:**
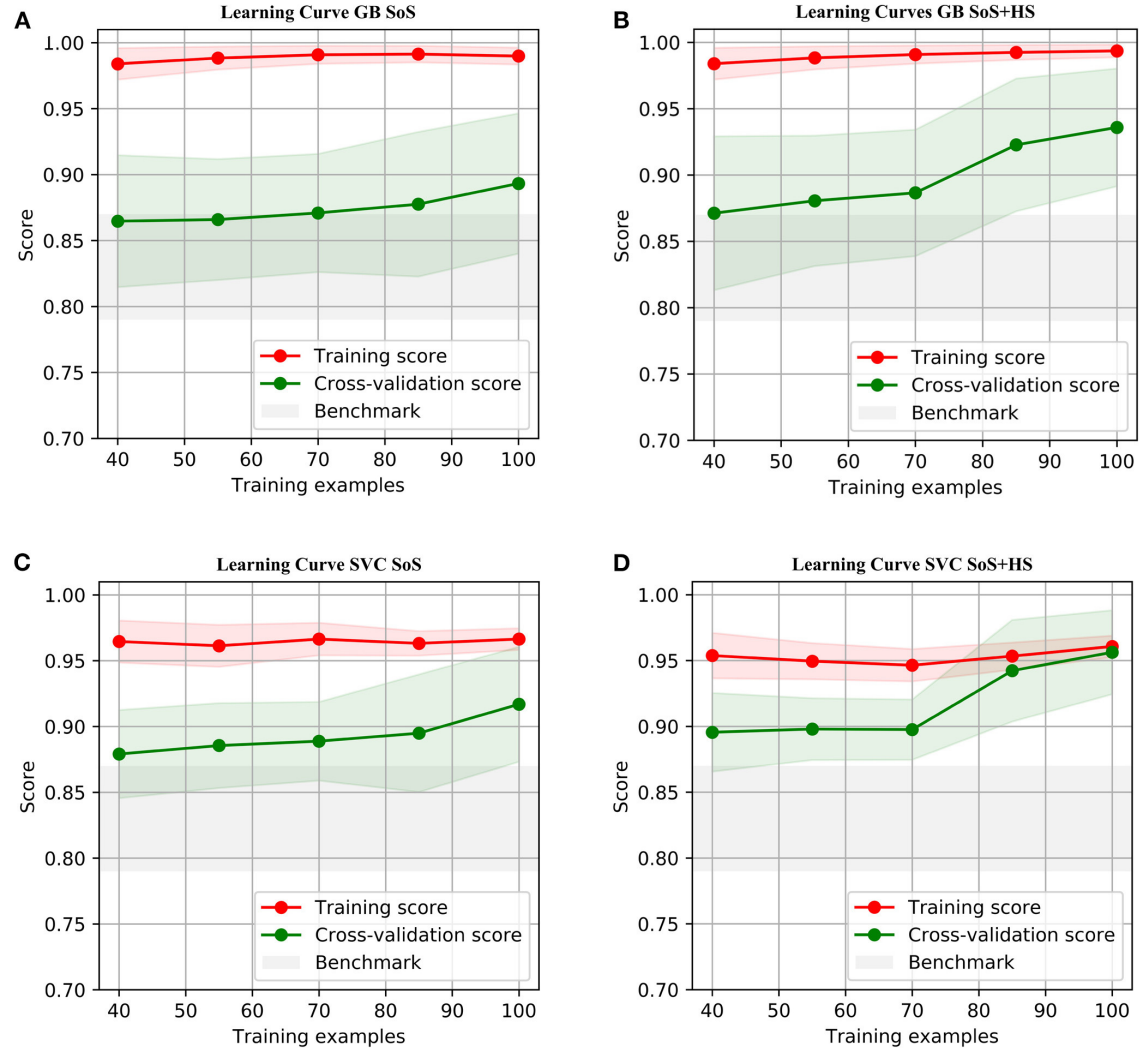
Learning Curves using accuracy score, with standard deviations. The test-fold accuracies (in green) are more representative of model performances on prospective data, showing enhanced learning by combining semiology and HS. **(A,C)** SoS has limited test-fold learning (green) with increasing training samples. **(B,D)** SoS+HS improves test-fold accuracies after about 70 samples. See Supplementary Materials for comparison with logistic regression.

**Figure 2:**
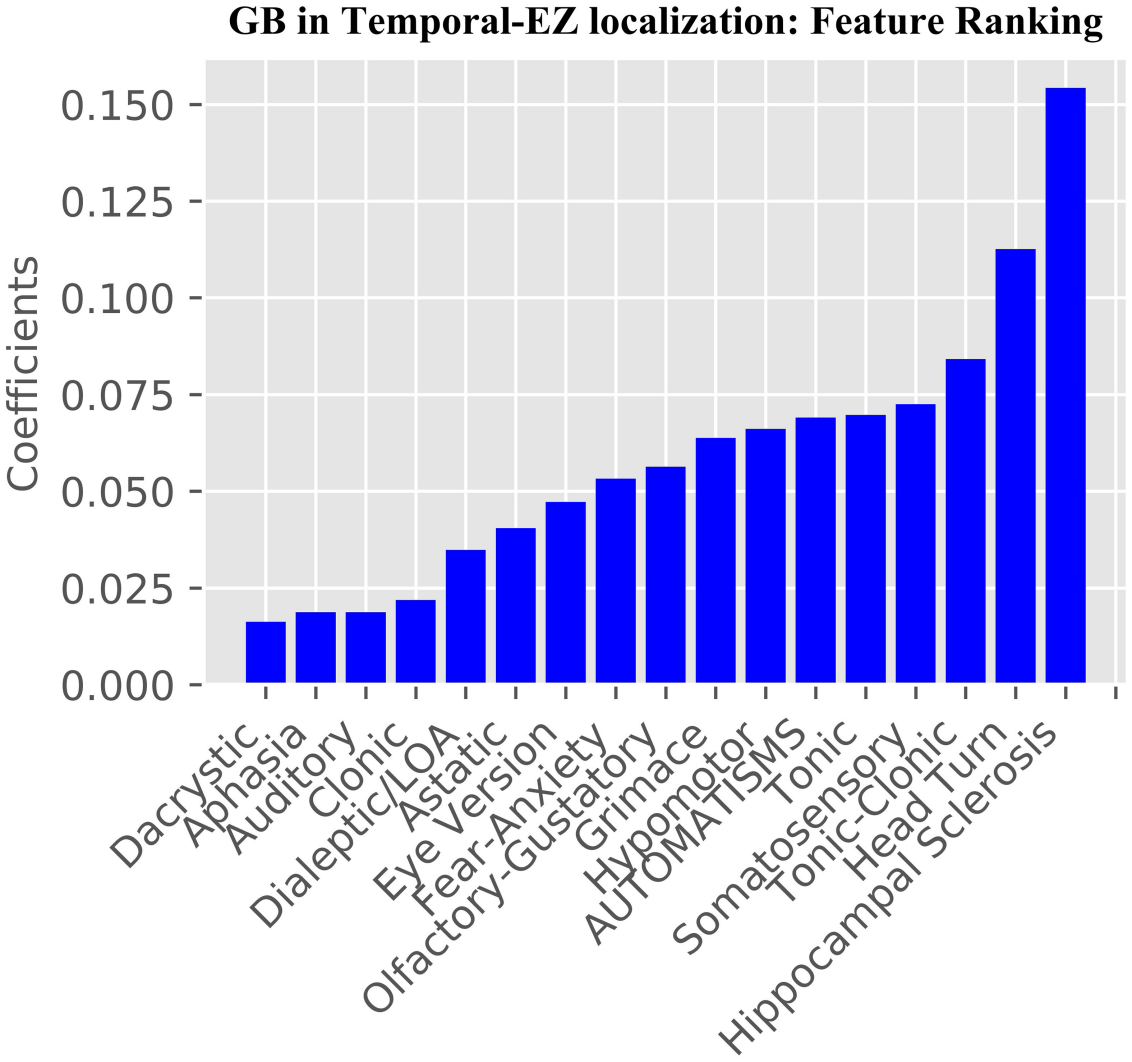
Gradient Boosting Classifier GB SoS+HS Feature Importance. From the 41 combined features, RFECV was used to determine the most relevant features for the model.

**Figure 3:**
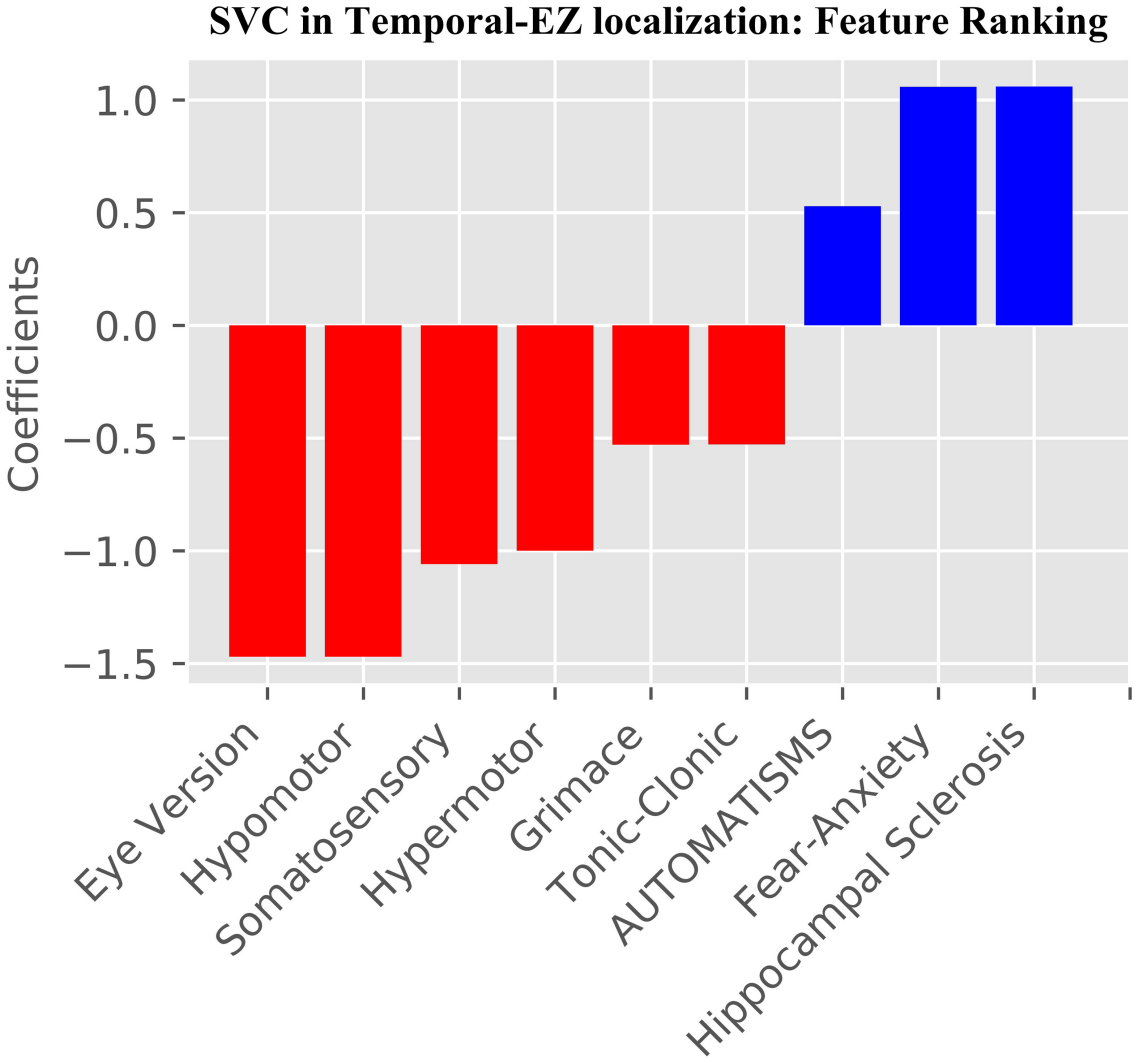
Support Vector Classifier SVC SoS+HS feature ranking using RFECV. In blue are features which predict temporal, and in red extratemporal EZ. All SVC features are also used by the GB model, except “Hypermotor” semiology.

**Figure 4:**
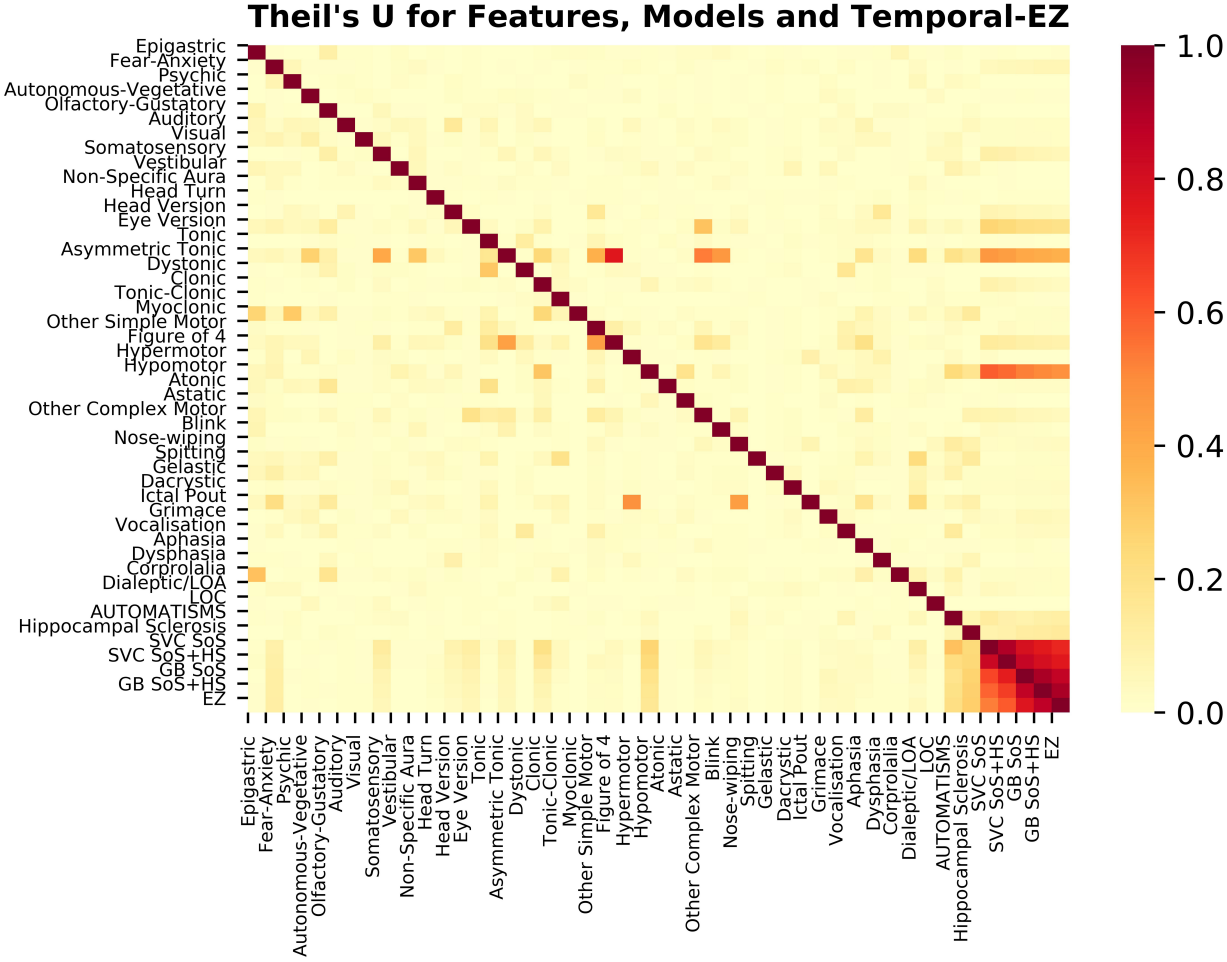
Theil's U for features, models and temporal-EZ localization. The columns represent the known (Bayesian prior) variable, and the rows the target entropy coefficient. For example, “Figure of 4” semiology in the column is a subset of “Asymmetric Tonic” in the row with high coefficient, whereas the reverse association is smaller in magnitude. The naïve algorithm would show zero association with all the variables with a column of zeros (and is undefined in row due to division by zero). All four models are more predictive of a temporal-EZ than any of the univariate features, with 1 (intense red) representing 100% of the information in the target being predicted by the column. With the addition of Imaging HS to Semiology SoS (*n* = 126, refit to training set) SVC and GB show a graded improvement in the proportion EZ-localization entropy accounted for.

The learning curves for the GB and SVC models show overfitting for SoS features alone that improved with combined SoS+HS features (Figure 1). Table 3 shows semiology and imaging enhanced performance above that of benchmarks using the best features obtained from RFECV (Figures 2, 3), most of which were found in the univariate analysis (Table 2). Figure 4 shows that combined features also enhance training-set performance.

GB betters SVC when refit to the ESF-set (Figure 4); whereas cross-validated results (Figure 1, Table 3) show the models perform more similarly: mean and median MCC with and without the imaging feature are:

- Best benchmark (imaging-HS): mean = 0.38 ± 0.08, median = 0.38, IQR = 0.33–0.43
- GB-SoS: mean = 0.36 ± 0.30, median = 0.35, IQR = 0.0–0.55
- GB-SoS+HS: mean = 0.64 ± 0.27, median = 0.66, IQR = 0.55–0.80
- SVC-SoS: mean = 0.48 ± 0.32, median = 0.55, IQR = 0.34–0.69
- SVC-SoS+HS: mean = 0.73 ± 0.25, median = 0.80, IQR = 0.55–0.80.

Comparing GB and SVC-models:

- with semiology alone, although SVC performed better, the two models performed similarly with overlap of interquartile ranges.
- with SoS+HS, there was also significant overlap between the models; the SVC-model again had a better median MCC.

Compared to SoS alone, when combining features:

- SVC mean, median, lower and upper quartiles were enhanced by between 10 to 25%. This suggests the support vectors are better defined with HS and that temporal lobe EZ are linearly separable in binary semiology-HS feature space.
- in the GB-model, there was also significant improvements in lower-quartile (55%), median (30%) and upper-quartile (25%) MCC and no overlap in interquartile ranges.
- LR (Table 3) shows similar improvements in metrics, except the median MCC remains at 0.55.

These affirm the value of combining multimodal features, irrespective of the model.

### Step 2: Indirect Surgical Outcome Results

Of the 183 NSF patients, 144 had temporal resections (54 ILAE 1 at 1-year, median of patient ILAE outcome medians = 2, IQR = 1–4) and 39 extratemporal resections (seven ILAE 1 at 1-year, median = 4, IQR = 2–4). Temporal resections were associated with better outcomes at 1-year post-resection (ILAE 1, OR = 2.7, *p* = 0.035) and better median ILAE outcomes (Mann-Whitney *U* = 2,057, *p* = 0.004). None of the machine learning models' congruent predictions with actual resections were significant in improving upon this naïve benchmark (Supplementary Figures 10–13).

### Step 3: Direct Surgical Outcome Results

Although direct (*n* = 309) benchmarks for ESF included having had a temporal-resection (OR = 2.2, *p* = 0.02), having been seizure-free-at-1-year, presence of HS (OR = 1.7, *p* = 0.02), and dysphasia (OR = 0.53, *p* = 0.039), and benchmarks for predicting seizure-freedom at 1-year included presence of HS (OR = 1.9, RR = 1.29, *p* = 0.005), temporal-lobe-resection (OR = 2.8, *p* = 0.001) and presence of intracranial EEG (OR = 0.46, *p* = 0.003), only seizure-freedom-at-1-year as a predictor of ESF was statistically significant after multiple comparisons correction (Theil's *U* = 0.43). No model was able to exceed naïve or feature benchmarks on any metric.

## Discussion

Our main findings were that models localized the epileptogenic-zone to the temporal lobe when using multimodal semiology and MRI report of HS, and were better than semiology, HS or other benchmarks in isolation. Support vector machines had a slight edge over Gradient Boosted trees, but there was considerable overlap in performances (Step 1). No method was able to predict seizure-freedom at 1-year or ESF better than benchmarks (Steps 2 and 3). Multicenter case records are required to confirm generalizability, and expanded features are necessary to determine if epilepsy surgical outcomes can be predicted at all.

### EZ-Localization Algorithms (Step 1)

Our study addresses a subset of the open issue of algorithmic identification of EZ networks (10), namely temporal-EZ, and provides univariate and algorithmic benchmarks with single (SoS) or two-modalities (SoS and HS). Models with multimodal features outperform semiology-only models (Figure 1) and univariate benchmarks (Table 3) using features that are significant on univariate analysis (Table 2) and those that are not (Figures 2, 3). The strength of the GB model lies in its ability to combine an ensemble of weak-learners, and out-perform individual univariate benchmarks, including the strongest, HS, as assessed on both training-set (Figure 4) and CV-folds (Table 3). SVC strength lies in classifying temporal-EZ by defining borderline cases as class-dividing support vectors. Support vectors are the feature-states of the cases which lie at the margins of the optimum hyperplane separating the temporal vs. extratemporal EZs. The SVC-model has 26 support vectors which determine the classifiers hyperplane. Alterations to any of these cases, but not others, can result in a different SVC classifier altogether. This makes the algorithm more robust to slight sample changes during cross-validation. The coefficients in Figure 3 represent the projections of a vector orthogonal to the classifying hyperplane onto each feature (15).

### Clinical Features of Temporal-EZ (Step 1)

The following cardinal semiologies of temporal lobe seizures have been described: (18)

- Prodromes
- Auras
- Altered Consciousness (dialeptic)
- Amnesia
- Automatisms (oral, manual, dacrystic, gelastic, and leaving-behaviors).

Hippocampal sclerosis is present in more than 80% of surgically treated TLE. The published semiologies in mTLE, commonly associated with HS include:

- Rising epigastric sensation
- Affective (fear)
- Experiential (including déjà vu)
- Automatisms
- Head Turns
- Autonomic phenomenon.

These semiologies are confirmed by univariate analysis (Table 2), and from the 17 retained features post-RFECV (Figure 2). A notable exception is rising epigastric sensation. Epigastric sensation is non-significant for the ESF patients used to train the data (Table 2) and not present as a feature after RFECV for either the SVC or GB models (Figures 2, 3).

There are conflicts and overconfidence in reporting the localizing values of semiology in the literature, using small samples of clinical cases and often no ground-truths to objectively assess labels or effects on surgical outcomes. The localizing values of semiologies may be stated without measuring confidence or variation e.g., postictal cough localizing to the temporal lobe (18), unilateral upper-limb automatisms reported to both have an ipsilateral seizure onset (19, 20) and no lateralizing value in isolation (21). Such discrepancies may arise due to lack of ground-truths, small numbers, ignoring time to onset of the semiology and excluding relevant features. When value is assessed, this is usually performed in a univariate manner, e.g., in one example series the trend that hypermotor seizures occur earlier in frontal lobe epilepsy than extra-frontal epilepsies was assessed by univariate Fisher's exact test, showing that chronology is valuable for EZ-localization; but did not reach significance and only 17 surgical patients were seizure-free (ground-truth labels), limiting the power of the analysis (22). The GB algorithm (Figure 2) shares all the SVC-model features (Figure 3) except hypermotor, which only features in the SVC-model, potentially making the SVC model more capable of identifying frontal-lobe (extratemporal) seizures.

### Quantifying Value of Multimodal Features

Although studies that look at single modality data can quantify the value of semiology compared to naïve benchmarks, they cannot assess the value of multimodal features, as are utilized clinically in MDTs (9). Clinical, demographic, imaging and neurophysiological features applied in machine learning have been purported to be capable of predicting mTLE outcomes (with or without HS), but this value has not been quantified nor applied to EZ-localization (13). Multimodal features of EEG and semiology enhance EZ-lateralisation accuracy (23), and although it is known that integration of clinical data also enhance EZ-localization (20), datamining studies have not quantified the incremental value of multimodal data (13).

Different methods may be used to assess incremental multimodal value; for any given model, the convergence rate of the learning curve, choice of performance metric, and maximum or average performance. We highlighted the value of semiology and imaging using all of these methods, and used suitable summary metrics in unbalanced datasets, MCC and NMI (Table 3). In both the GB SoS+HS and SVC SoS+HS models, multimodal features improve MCC and NMI average scores by over 25% compared to the best univariate benchmark of HS, and compared to the SoS-only models. Therefore, although SoS is not more valuable than univariate markers, when combined with the imaging feature (HS) it enhances epileptogenic lobe localization.

### Outcome Prediction (Steps 2 and 3)

In Step 2 we evaluated model performance in indirectly predicting outcomes on the 183 non-seizure free patients. We assessed the veracity of these EZ-labels using the model as the predictor of true labels. The null hypothesis was that if there was a mismatch between the actual resection (weakly labelled EZ) and prediction, the ILAE outcomes should not be significantly different to when there is congruence of prediction. A naïve benchmark which predicts all resections to be temporal outperforms models from Step 1, therefore the EZ-localization performance does not translate to better outcomes.

Step 3 directly used all 309 patients' features to predict seizure-freedom, and the training curves showed overfitting as the models performed much better on the training set, but were no better than benchmarks on cross-validation folds (Supplementary Figure 12). Features which could localize temporal-EZ within the context of the above algorithms are thus insufficient for outcome prediction, which limits their clinical utility (8). Many other factors besides the EZ may determine outcomes, including whether there are indicators of multifocal epilepsy, unaccounted clinical (24) and genetic features, lesion histology (25), EEG patterns, and extent of surgical resection (11, 26–29). Our model did not account for these, nor the precise structures within the temporal lobe that were resected.

Table 2 suggests that invasive EEG is more likely to be used in extra-temporal-EZ, but is not associated with better outcomes, reflecting selection bias, in that invasive EEG would only be used if localization was unclear on non-invasive investigations.

We were not able to predict outcomes with our chosen features using GB, SVC, or other models, as reported previously (30). However, other studies have purported to be capable of predicting mTLE binary post-surgical outcomes using various models and features in cross-validated studies: naïve-Bayes and SVC (max accuracy 95%) (13), neural networks and wide manual data abstraction (accuracy 92%); neural networks and diffusion-tensor imaging (PPV of 88 ± 7%) (31, 32). The smaller studies are likely to be overfitting the data and not generalizable, and even accurate prognostication does not help improve clinical outcomes (33).

### Limitations

The mean CV score is considered an unbiased estimate of performance. The standard deviation estimates for the CV scores are however not unbiased (34); these are particularly large due to different training samples within each fold (e.g., SVC is sensitive to the support vector cases), and some folds predicting no extratemporal EZs due to class imbalance, resulting in larger variances for NPV and specificity (Table 3). As we tuned the number of features using RFECV, the mean CV score is also biased, therefore multicenter prospective data is required to assess generalizability and ascertain which model is inherently more suited to localizing temporal-EZ. The learning curves also suggest further data may enhance results.

We used the complete set of available ictal symptoms and not only the semiology presenting at seizure-onset or a sequential Markov model, which together with omitted imaging, electrophysiological and neurophysiological features may yield better results.

We did not model propagation networks in which similarly located lesions may differentially straddle inherent brain networks. Dichotomous assumption of temporal vs. extra-temporal lobe epilepsy may be only good insofar as the majority of resections are anterior temporal resections. Our labels do not differentiate between lateral or mesial temporal-lobe EZ or indeed the extent of resection.

The PPV and specificity of both semiology and HS are higher than the models in predicting temporal-EZ, although the training-scores are comparable. The GB SoS+HS model has a more balanced metric profile, as reflected in F1-macro, MCC and NMI scores (Table 3).

A strength of our study is the inclusion of only patients who remained ESF for epileptogenic zone localization, despite the good results for localization, this doesn't translate to better outcomes, the so-called AI chasm is thus not surmounted.

Further work is required to validate this localization model prospectively. Expanding the number of training samples and features in a multicenter approach may allow the use of these models to localize epileptogenic networks to a greater level of detail, and allow investigation of the extent that surgical outcomes can or cannot be predicted with all available multimodal data.

## Data Availability Statement

Due to patient confidentiality, the datasets are not publicly available, but anonymised versions can be made available upon reasonable request.

## Ethics Statement

This study was approved by the Research Ethics Committee for UCL and UCLH (20/LO/0149).

## Author Contributions

AA-M designed the study, wrote the code for health record pre-processing, data mining and data analysis, performed the statistics, trained machine learning models, made inferences, obtained funding, and wrote the manuscript. FP-G edited the manuscript and checked results. KD trained machine learning models and checked results. GR, SO, and RS edited the manuscript. BD was involved in devising semiology features and edited the manuscript. MC edited the manuscript and supervised the study. JD conceived the research programme, designed the study, obtained funding, edited the manuscript, and supervised the study. All authors contributed to the article and approved the submitted version.

## Conflict of Interest

The authors declare that the research was conducted in the absence of any commercial or financial relationships that could be construed as a potential conflict of interest. The reviewer GS declared a past collaboration with one of the authors AA-M.
