# Supplementary material for: Machine Learning for Localizing Epileptogenic-Zone in the Temporal Lobe: Quantifying the Value of Multimodal Clinical-Semiology and Imaging Concordance
Source: Front Digit Health. 2021 Feb 10;3:559103. doi: 10.3389/fdgth.2021.559103 (PMC8521800; doi:10.3389/fdgth.2021.559103)
Supplement: Supplementary file 1 [file Data_Sheet_1.docx]

# Supplementary Materials

## Machine Learning models and default parameters

All models were imported from scikit-learn version 0.19.2 with default settings

- GradientBoostingClassifier()
- LogisticRegression()
- SVC(kernel=”linear”)
- RandomForestClassifier()
- BernoulliNB()

Random state was set to 1 for RFECV and for evaluation.

- RFECV(model, step=1, cv=StratifiedKFold(5, **random_state=1**))
- rskf = RepeatedStratifiedKFold(n_splits=5, n_repeats=1000, **random_state=1**)
- Cross_val_score(model, X[best_features], cv=rskf)

## Feature Evaluations


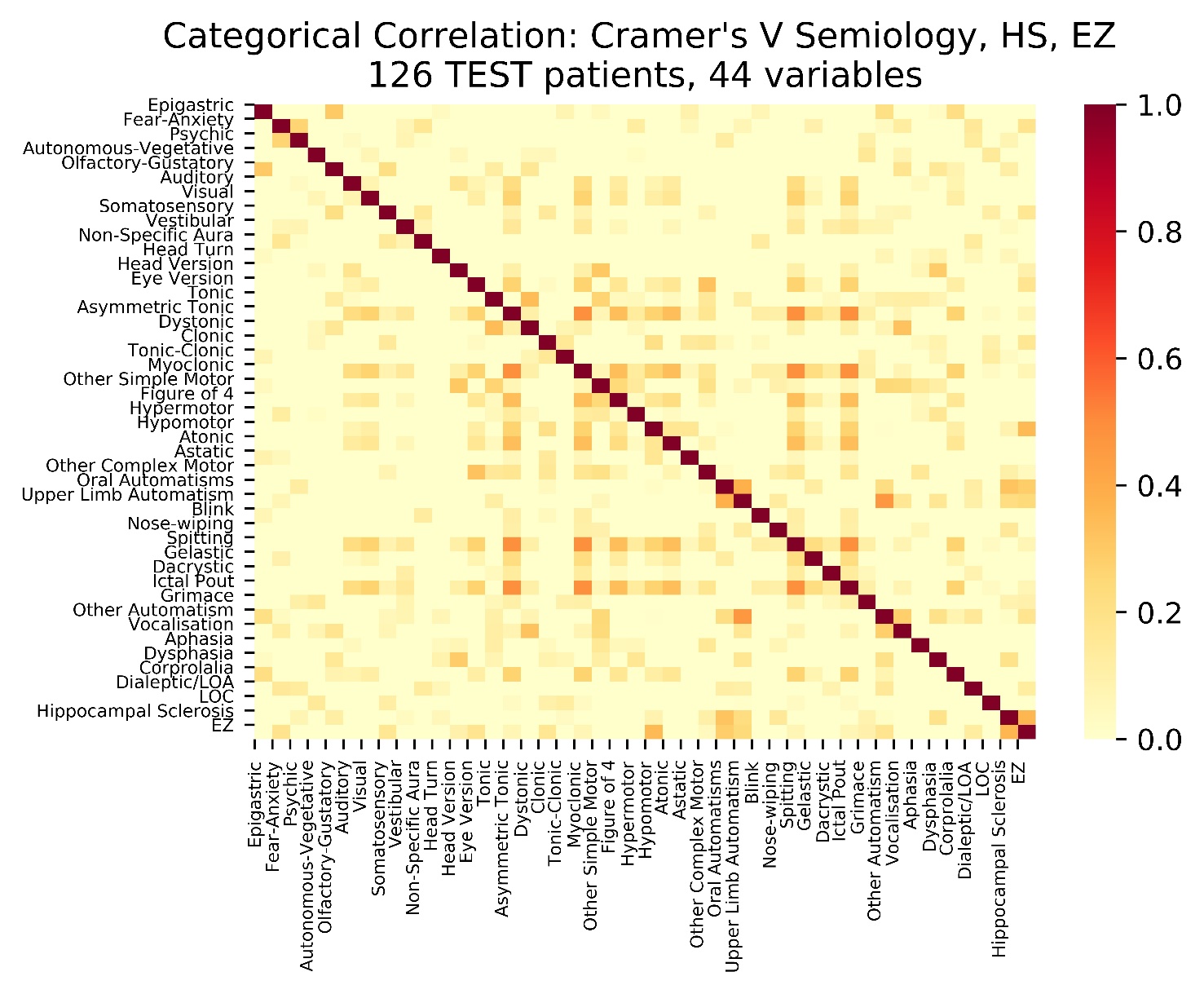


Figure 1: unmerged categories of semiology features, Cramer’s V. Note that these are symmetric univariate categorical associations, where EZ is correlated to HS, hypomotor (behavioural arrest), oral automatisms and fear-anxiety.

## RFECV: Recurrent Feature Elimination Cross-Validation

A
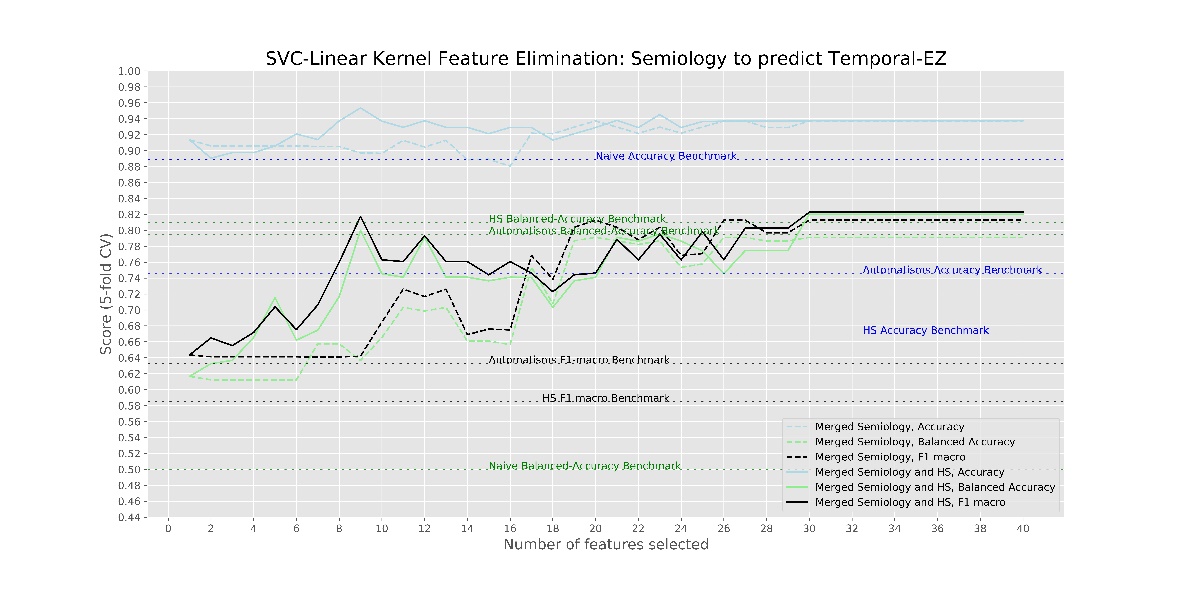


B
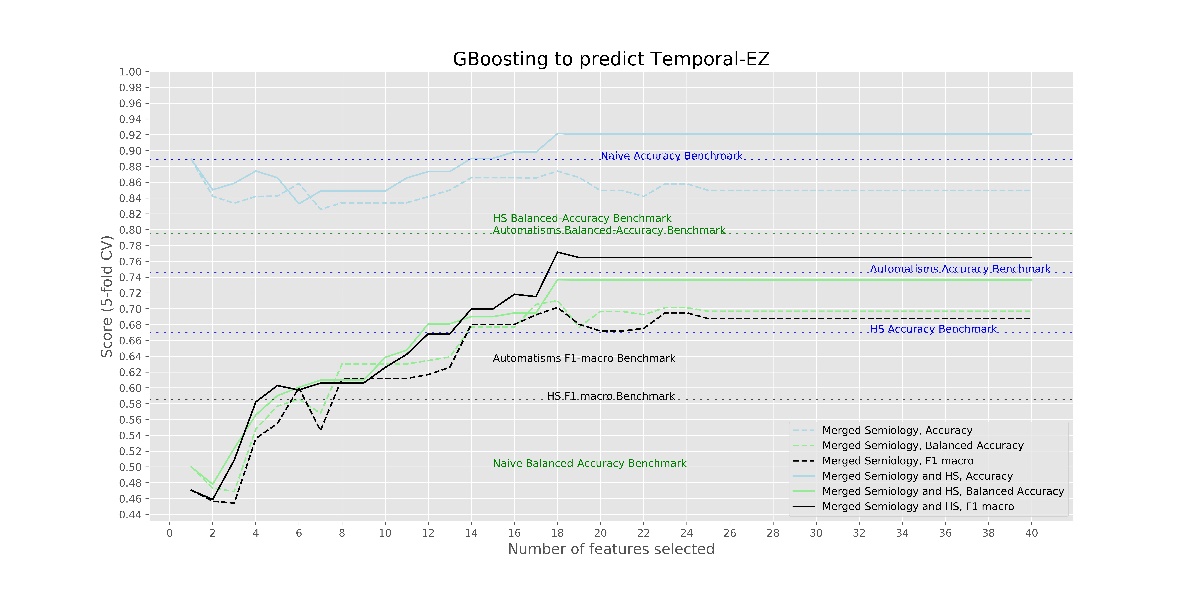


C
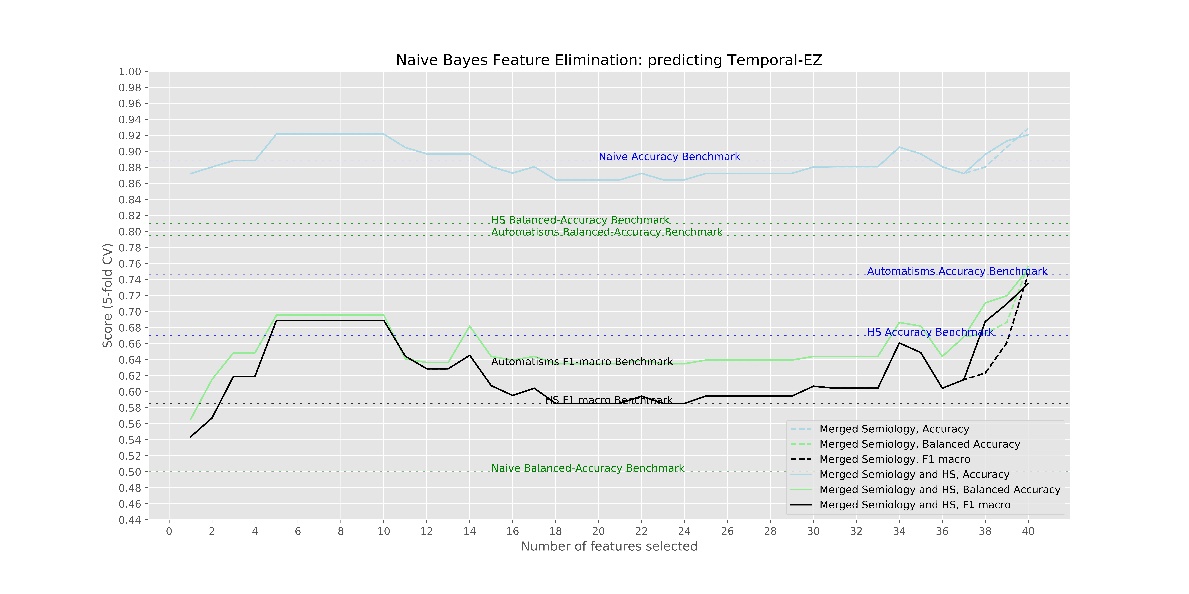


Figure 2: RFECV accuracy, balanced accuracy and F1-macro score plots against number of features for (**A)** SVC,(**B)** GB and (**C**) NB classifiers. The benchmark scores are dashed horizontal lines.


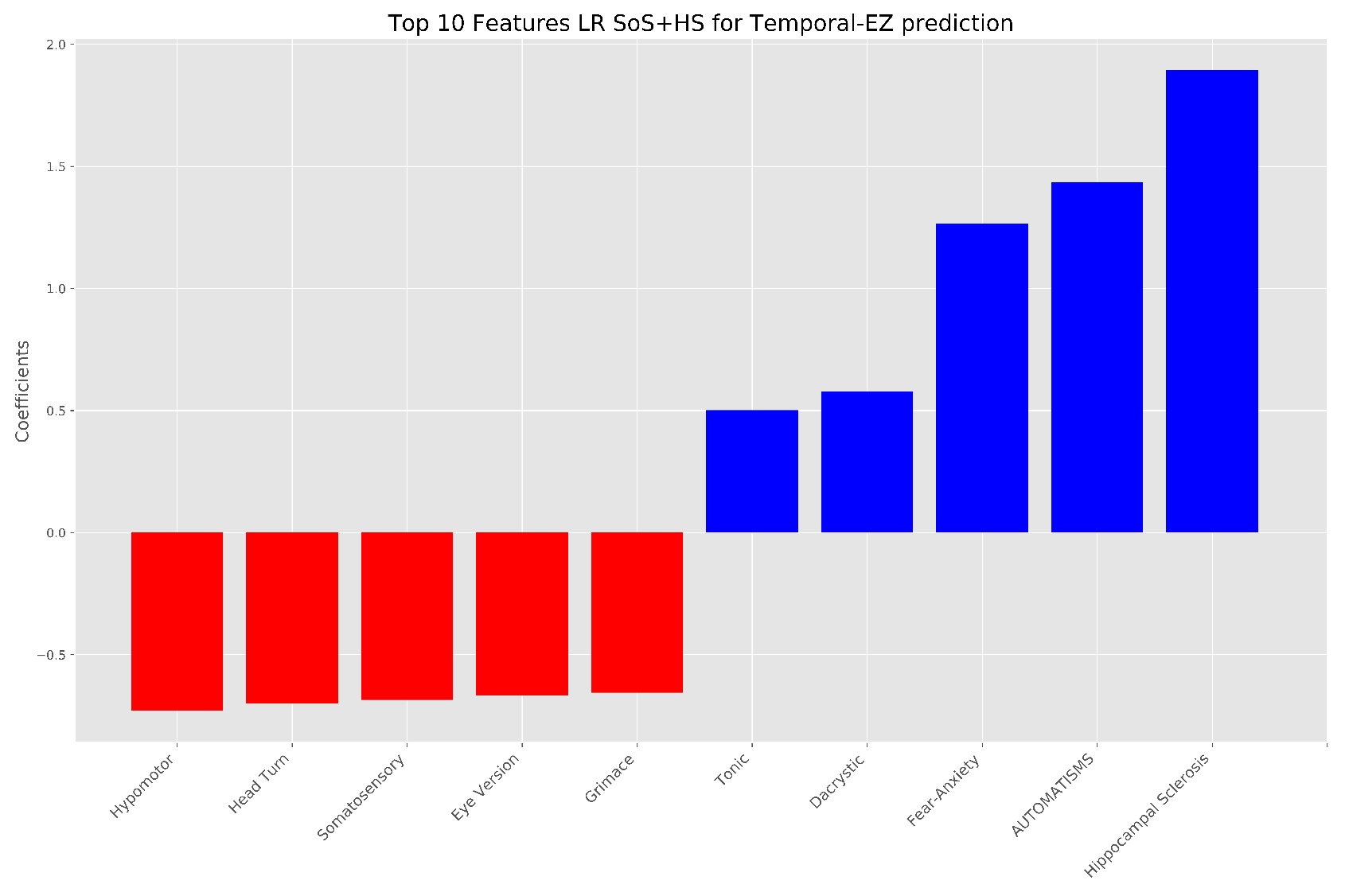


Figure 3: The best features obtained from RFECV for LR SoS+HS model, which was not presented in the manuscript. The features are very similar to the ones obtained from GB and SVC and included in the manuscript.

## Learning Curves

The learning curves for GB and linear-SVC after RFECV are in the main manuscript.

The learning curves for SVC, LR with and without HS and with and without RFECV are presented below.


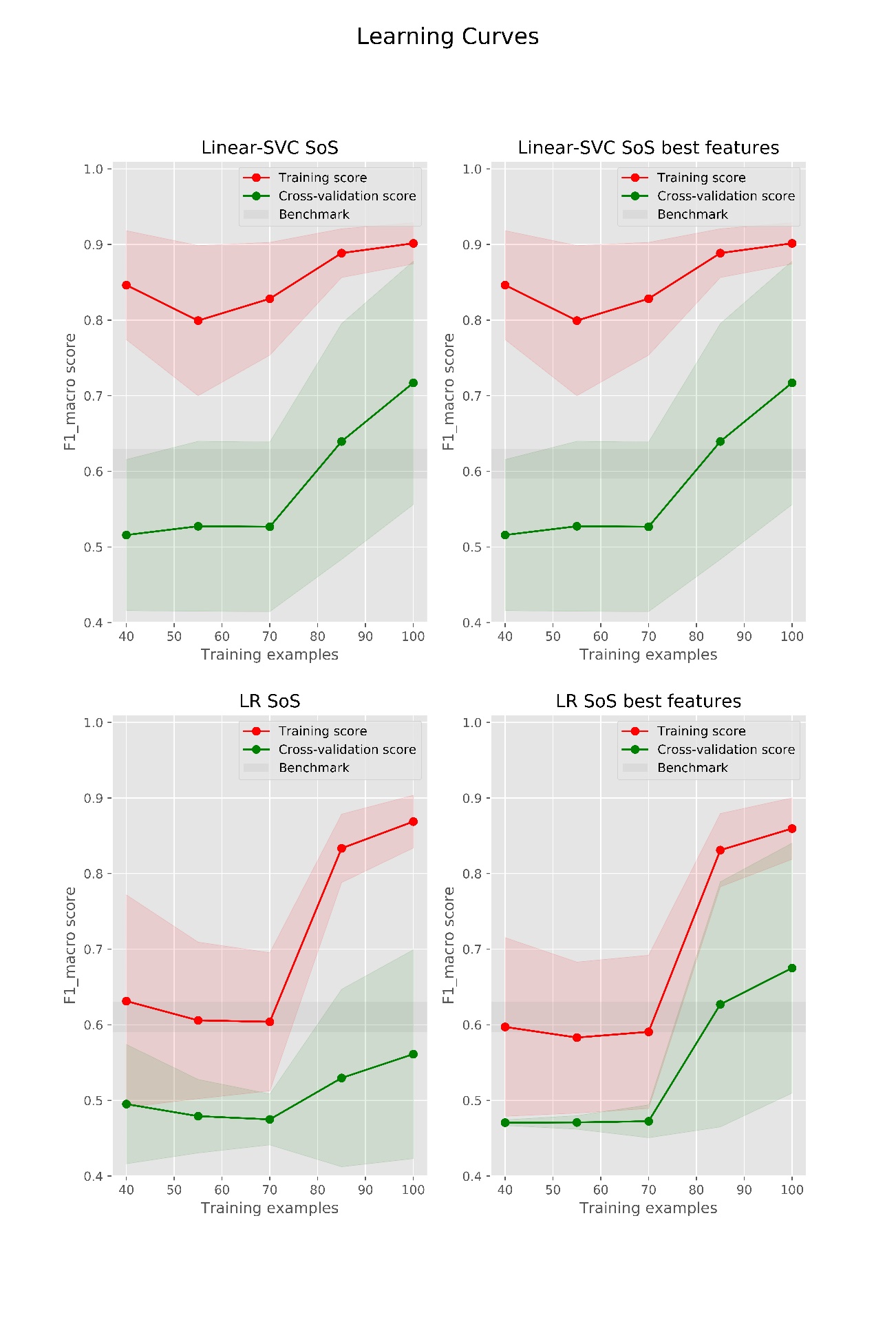


Figure 4: learning curves, F1_macro score against training samples for linear-SVC and LR models. The panel on the left shows learning curves using all features. The panel on the right shows the learning curves using only the best features obtained from RFECV. Comparing the validation set scores (green) from RFECV shows similar or better results.

A
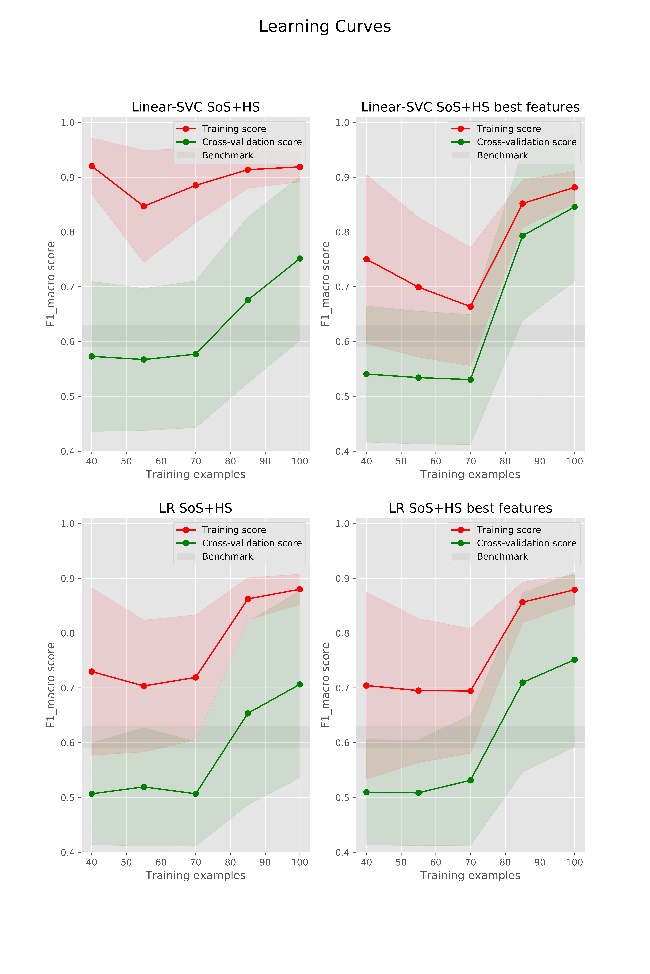
B
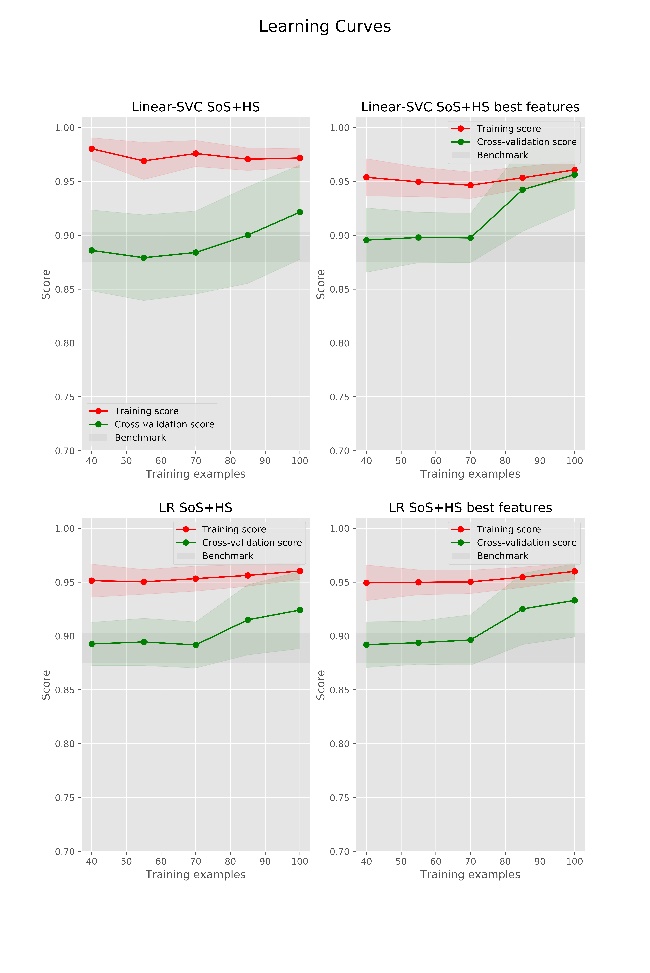


Figure 5: learning curves for SVC and LR SoS+HS, (**A**) F1_macro against training samples. (**B**) accuracy score against training samples. RFECV improves SoS+HS validation fold results. Validation fold results are generally better than when using SoS alone, as in Figure 4.

## **Expanded Manuscript Table 3 Machine Learning Models for Temporal EZ-Localisation (Step 1)**

| **Model-RFECV**  **5-CV**  **metric**  **+/-std (refit)** | **Naïve**  **Benchmark** | **Automotor**  **Semiology**  **Univariate**  **Benchmark** | **HS**  **Imaging**  **Univariate**  **Benchmark** | **LR SoS** | **LR SoS+HS** | **Linear Support Vector Classifier SoS** | **Linear Support Vector Classifier SoS+HS** | **Naïve Bayes Bernoulli (SoS/ SoS+HS)** | **Random Forest**  **SoS** | **Random Forest**  **SoS+HS** | ***GB**  **SoS+HS** |
| --- | --- | --- | --- | --- | --- | --- | --- | --- | --- | --- | --- |
| **# of features (min equivalent)** | N/A | 1 | 1 | 16 | 25 (18) | 40 (30) | 9 | 41 (5) | 22 (7) | 21 (11) | 17 |
| **F1 average macro** | N/A | 0.61± 0.06 | 0.59± 0.06 | 0.68 ± 0.17 (0.88) | 0.75 ± 0.16 (0.88) | 0.72 ± 0.16 (0.88) | 0.85 ± 0.14 (0.91) | 0.76 ± 0.15 | 0.60 ± 0.15 (0.98) | 0.68 ± 0.16 (0.98) | 0.81 ± 0.14 (0.98) |
| **Balanced Accuracy** | 0.5 | 0.67± 0.07 | 0.75± 0.04 | 0.65 ± 0.13 (0.82) | 0.72 ± 0.15 (0.82) | 0.70 ± 0.15 (0.82) | 0.81 ± 0.14 (0.86) | 0.73 ± 0.14 | 0.60 ± 0.12 (0.96) | 0.67 ± 0.14 (0.96) | 0.80 ± 0.15 (0.96) |
| **Accuracy** | 0.83 ± 0.04 | 0.71± 0.05 | 0.63± 0.05 | 0.92 ± 0.03 (0.96) | 0.93 ± 0.03 (0.96) | 0.92 ± 0.04 (0.96) | 0.96 ± 0.03 (0.97) | 0.93 ± 0.04 | 0.89 ± 0.04 (0.99) | 0.91 ± 0.04 (0.99) | 0.93 ± 0.05 (0.99) |
| **Sensitivity / Recall** | 1 | 0.73± 0.06 | 0.56± 0.06 | 1.0 ± 0.004 | 0.995 ± 0.015 | 0.98 ± 0.03 | 1.0 ± 0.006 (1.0) | 0.98 ± 0.03 | 0.97 ± 0.04 (1.0) | 0.98 ± 0.03 (1.0) | 0.97 ± 0.04 (1.0) |
| **Specificity** | 0 | 0.62± 0.14 | 0.94± 0.06 | 0.30 ± 0.26 (0.64) | 0.44 ± 0.29 (0.64) | 0.42 ± 0.29 (0.64) | 0.61 ± 0.28 (0.71) | 0.49 ± 0.28 | 0.12 ± 0.19 (0.93) | 0.25 ± 0.25 (0.93) | 0.62 ± 0.29 (0.93) |
| **PPV** | 0.83 ± 0.04 | 0.90± 0.04 | 0.98± 0.02 | 0.92 ± 0.03 (0.96) | 0.94 ± 0.03 (0.96) | 0.93 ± 0.03 (0.96) | 0.96 ± 0.03 (0.97) | 0.94 ± 0.03 | 0.91 ± 0.03 (0.99) | 0.92 ± 0.03 (0.99) | 0.95 ± 0.03 (1.0) |
| **NPV** | 0 | 0.32± 0.09 | 0.31± 0.07 | 0.64 ± 0.48 (1.0) | 0.77 ± 0.39 (1.0) | 0.67 ± 0.40 (1.0) | 0.93 ± 0.25 (1.0) | 0.72 ± 0.36 | 0.26 ± 0.42 (1.0) | 0.51 ± 0.47 (1.0) | 0.76 ± 0.31 (1.0) |
| **AUROC** | N/A | N/A | N/A | 0.89 ± 0.11 | 0.95 ± 0.06 | 0.83 ± 0.14 | 0.95 ± 0.05 | 0.89 ± 0.10 | 0.73 ± 0.16 | 0.81 ± 0.15 | 0.95 ± 0.07 |
| **Average Precision** | N/A | N/A | N/A | 0.98 ± 0.02 | 0.99 ± 0.01 | 0.97 ± 0.03 | 0.99 ± 0.01 |  |  |  | 0.99 ± 0.01 |
| **MCC [bootstrap refit]** | 0 | [0.28± 0.12] | [0.38± 0.08] | 0.41 ± 0.33  [0.76 ± 0.22] (0.78) | 0.55 ± 0.31  [0.76 ± 0.22] (0.78) | 0.48 ± 0.32  [0.76 ± 0.22] (0.78) | 0.54 ± 0.29  [0.81 ± 0.19] (0.83) | 0.55 ± 0.29  [0.72 ± 0.23] (0.74) | 0.24 ± 0.30  [0.96 ± 0.09] (0.96) | 0.41 ± 0.31 [0.96 ± 0.09] (0.96) | 0.64 ± 0.27  [0.96 ± 0.09] (0.96) |
| **NMI symmetric CV**  **[asymmetric bootstrap refit]** | 0 | [0.10± 0.07] | [0.21± 0.08] (0.28) | 0.31 ± 0.26  [0.57 ± 0.29] (0.53) | 0.42 ± 0.28  [0.57 ± 0.29] (0.53) | 0.35 ± 0.28  [0.57 ± 0.29] (0.53) | 0.38 ± 0.27  [0.65 ± 0.29] (0.604) | 0.39 ± 0.27  [0.53 ± 0.29] (0.46) | 0.17 ± 0.21  [0.91 ± 0.19] (0.87) | 0.28 ± 0.25  [0.91 ± 0.19] (0.87) | 0.48 ± 0.29  [0.91 ± 0.19] (0.87) |

Expanded Manuscript Table 3: Machine Learning Models for Temporal EZ-Localisation (Step 1). Step 1 CV performance metrics. Mean and standard deviation of 1,000 x 5 CV scores. Benchmark std given by bootstrapping 2,000 x 5 CV. Brackets represent model-refit (training) scores. Square brackets bootstrap model refit samples. **CV**=cross-validation. **RFECV**=Recursive Feature Elimination with CV. **std**=standard deviation. **PPV/NPV**=Positive/Negative Predictive Value. **AUROC**=Area under receiver operating curve. **MCC**=Matthews Correlation Coefficient. **NMI**=Normalised Mutual Information.

## Value of Concordance: SoS vs SoS+HS Distribution of Scores (Step 1, EZ-localisation)


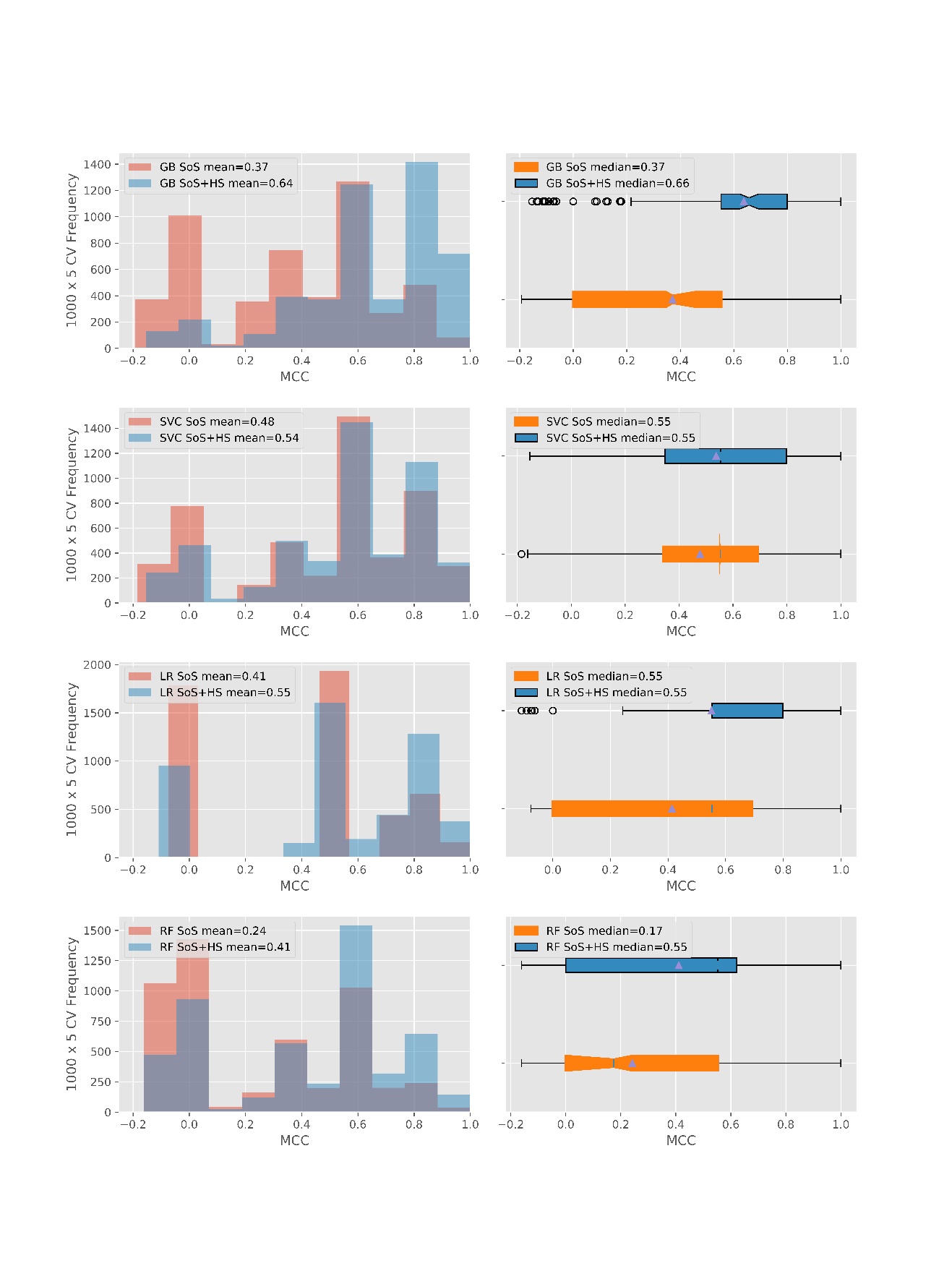


Figure 6: MCC distributions for SoS and SoS+HS results for GB, SVC, LR and RF. The panel on the left shows the distribution of MCC scores using 1000 x 5 CV, showing GB SoS+HS has the highest proportion of MCC=1.0. The right panel shows the boxplots, showing only in the case of GB was there a clear improvement in scores with concordance.


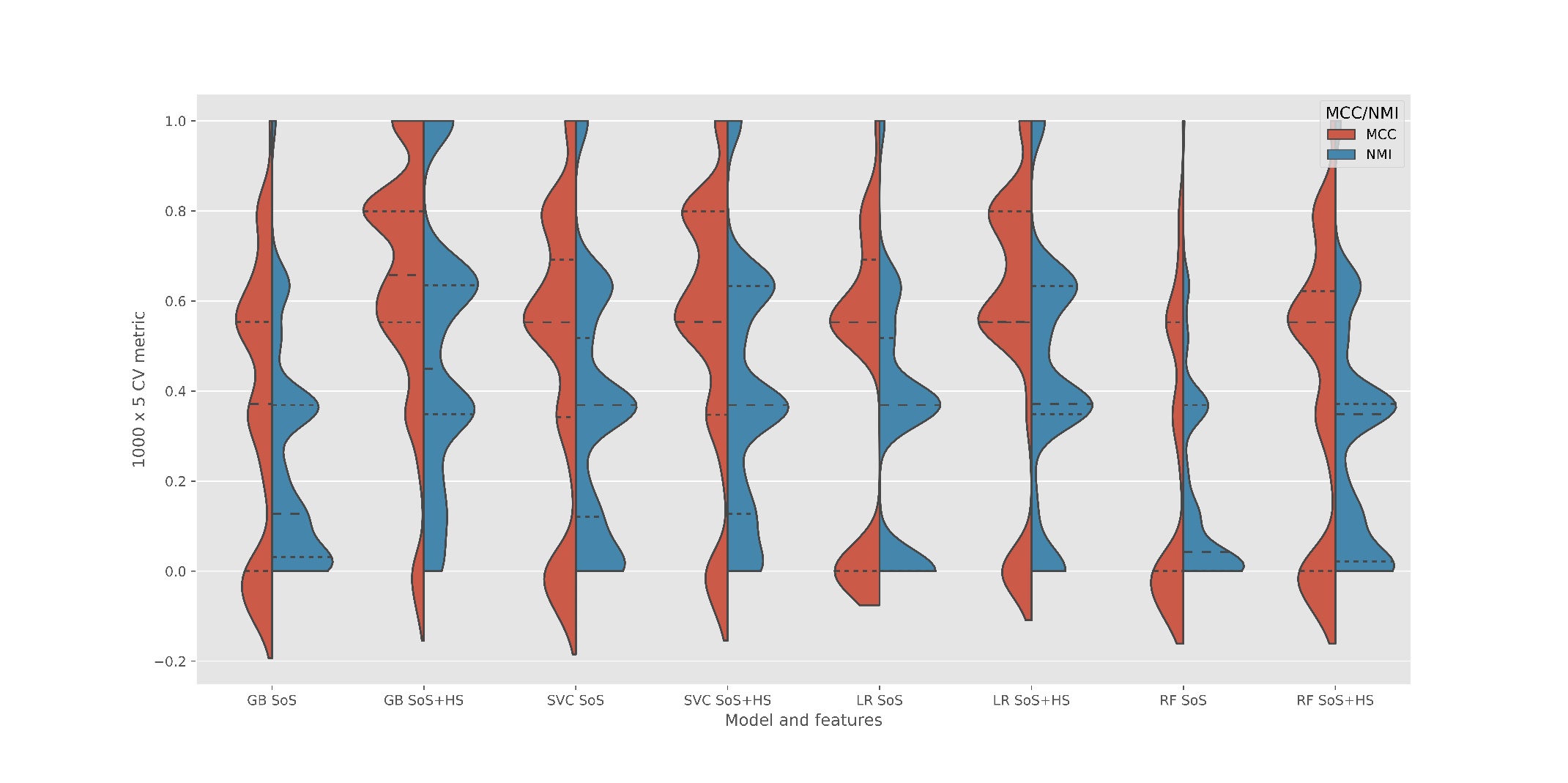


Figure 7: MCC and NMI scores for models. These violin plots show the distributions of the MCC scores in more detail than boxplots. MCC scores can lie between [-1,1] whereas NMI scores vary between [0,1]. LR SoS+HS performs well, but has lower median and less scores at 1.0 than GB SoS+HS.

## Unsupervised Methods


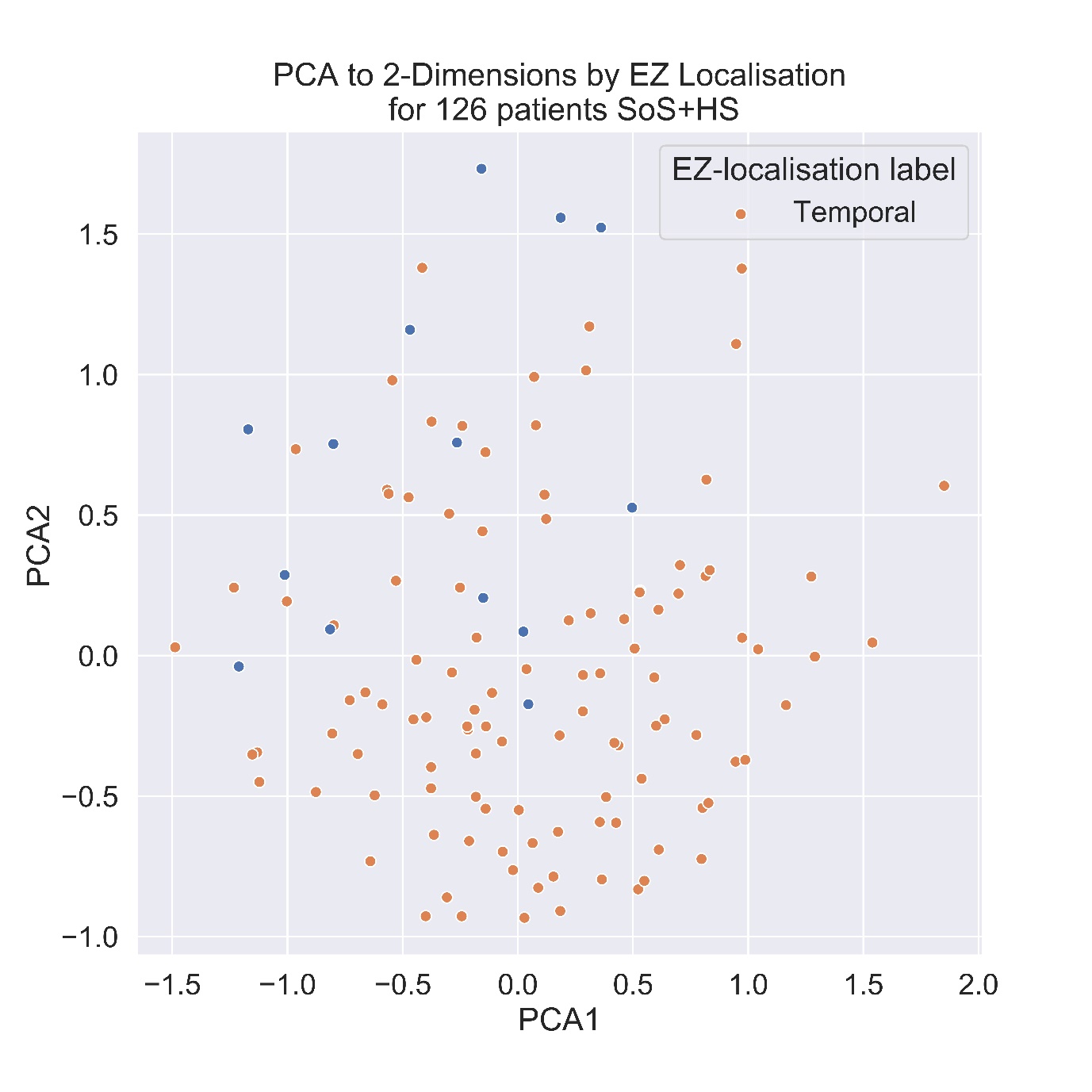


Figure 8: Principle Component Analysis of the training-set features SoS+HS, in 2-dimensions. The data was labelled for EZ. Higher polynomial orders (2 and 3) were not helpful either in segregating the labels. PCA was not helpful in feature reduction.

## Steps 2 and 3: Outcome Prognosis


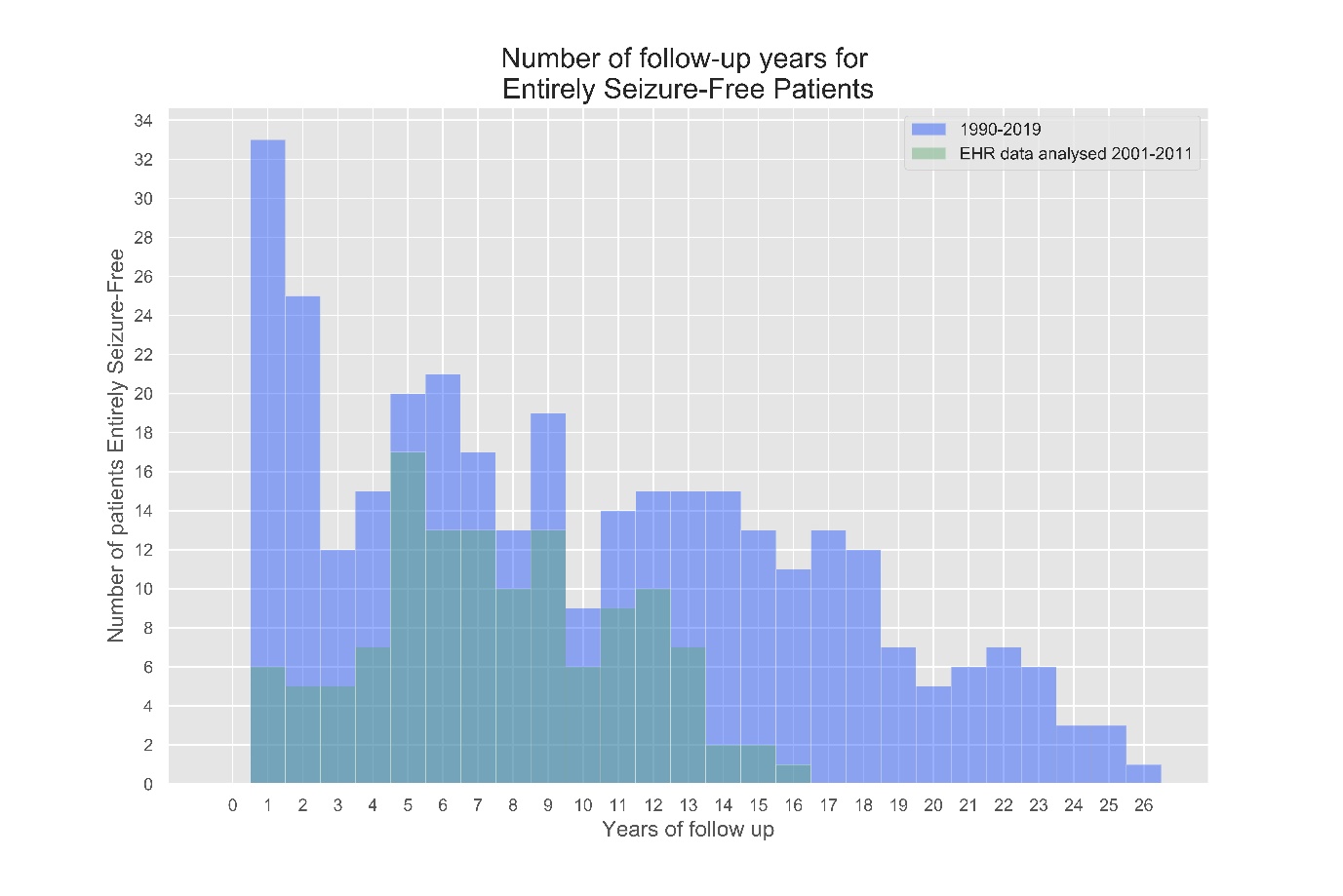


Figure 9: Frequency distribution of the numbers of years of follow-up for the 10 years of Electronic Health Record (EHR) data analysed (green) compared to the entire population of patients operated at in the centre from 1990. Median follow-up 7 years, IQR = 5–10.


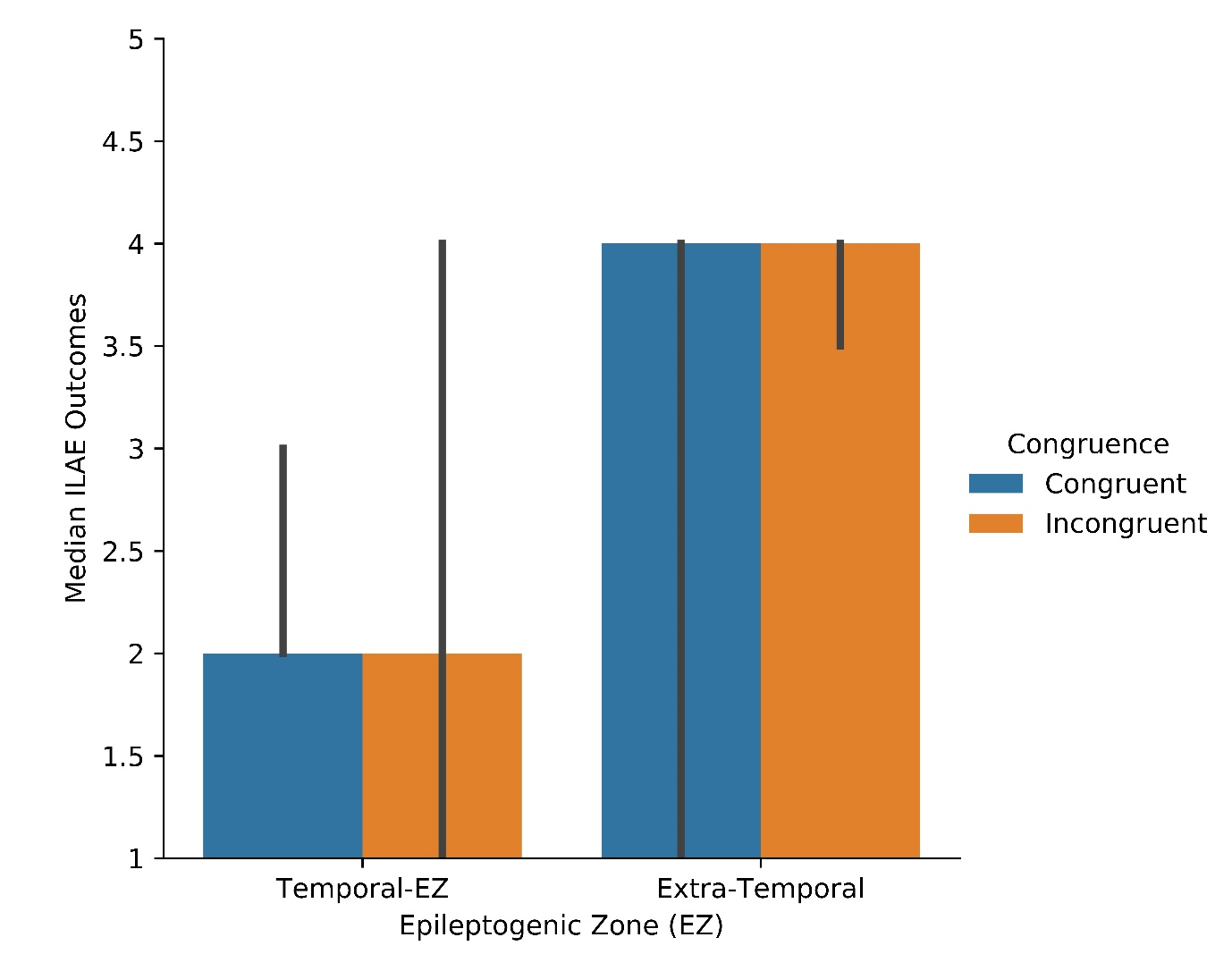


Figure 10: Median outcomes by congruence of model prediction to actual resected lobe for linear SVC SoS+HS. Bootstrapped 95% confidence intervals. Incongruent extratemporal resections fair worse than congruent temporal resections, but this is a subset of extratemporal cases generally having worse outcomes.


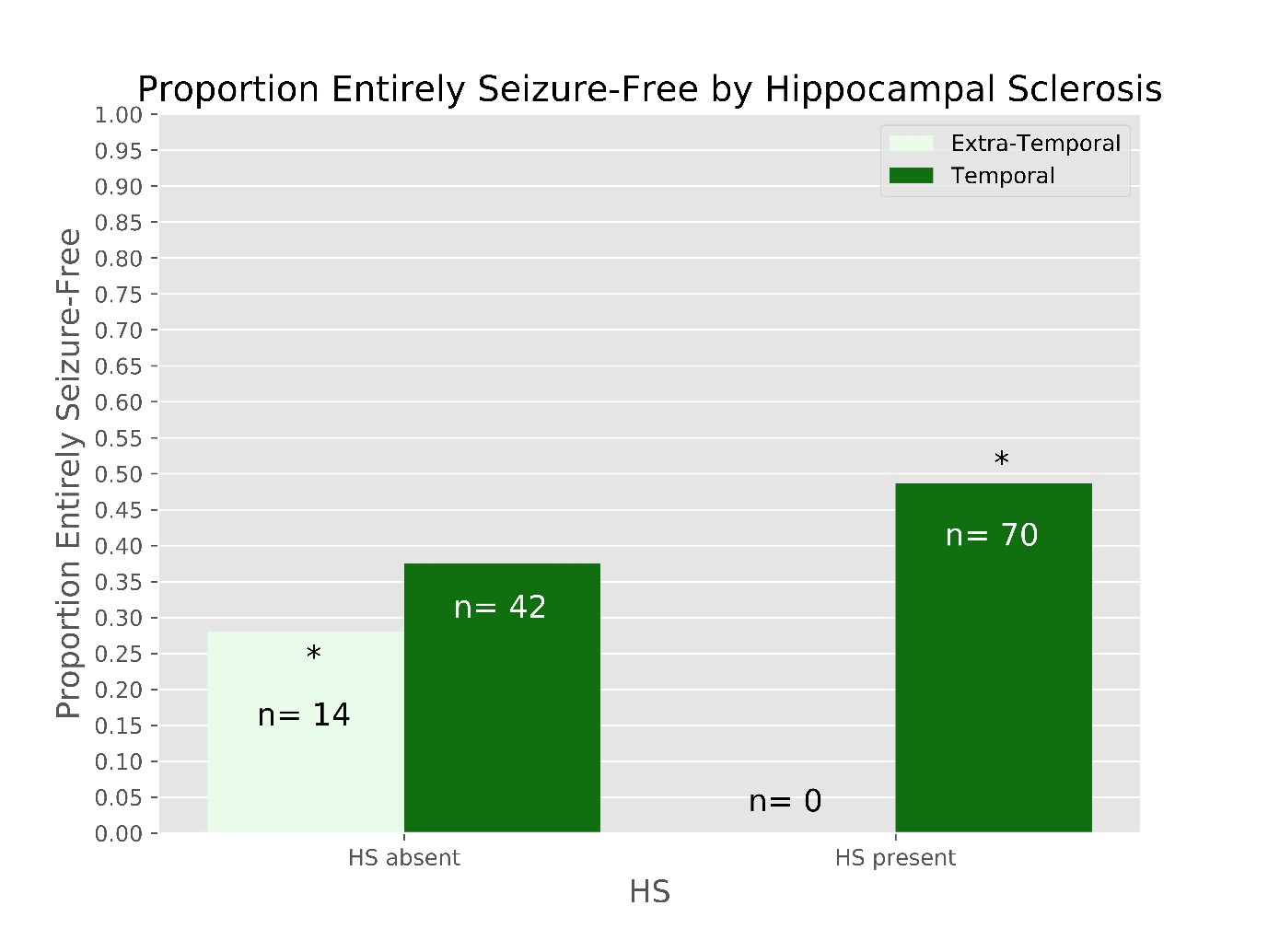


Figure 11: Interaction between imaging-HS and temporal vs extratemporal resections. At the alpha 5% level of significance, extratemporal resections without HS have worse ESF rates (two-by-two Fisher’s exact). On the other hand, patients with temporal resections and HS have better outcomes. Note that if instead of just the presence or absence of HS we use all imaging features of lesional epilepsy, then there are 6 extra cases lesion-present cases (on the right), and 6 less from the imaging-absent cases (on the left) and the statistical tests are unaffected.

We reported no model was able to predict outcomes better than benchmarks, this was despite also using the resected lobe label as an extra feature. Note the valuable interaction (Figure 12) between imaging/HS and Temporal-EZ resections.


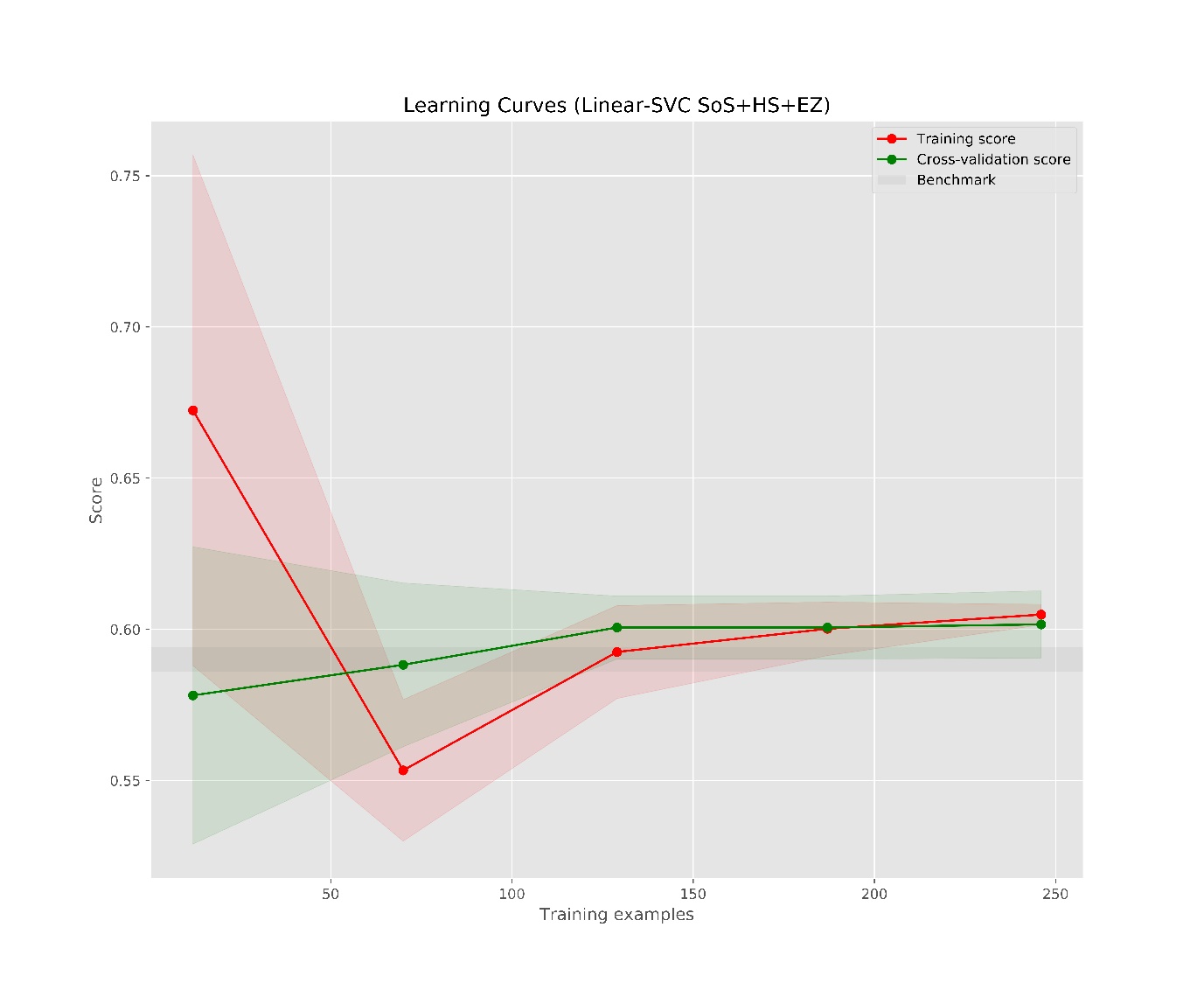


Figure 12: learning curve accuracy vs training samples for Step 3, prediction of outcomes (ESF), using linear-SVC with SoS+HS+EZ features. There is no learning above benchmark.


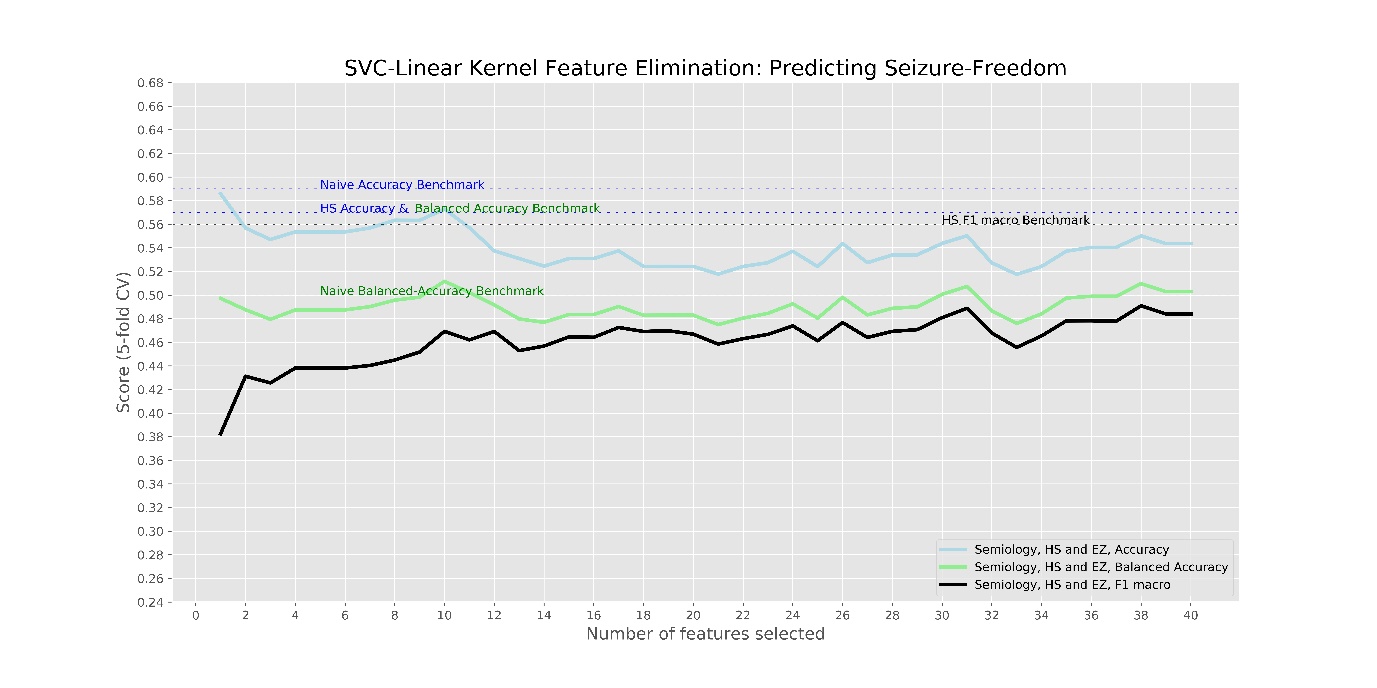


Figure 13: RFECV for SVC for the prediction of seizure-freedom (ESF). There is no significant improvement over benchmarks. Predicting seizure-freedom at one-year, instead of entirely seizure-free, produced similar results.
